# Integrating the Built and Social Environment into Health Assessments for Maternal and Child Health: Creating a Planning-Friendly Index

**DOI:** 10.3390/ijerph17249224

**Published:** 2020-12-10

**Authors:** Xi Wang, Jennifer Whittaker, Katherine Kellom, Stephanie Garcia, Deanna Marshall, Tara Dechert, Meredith Matone

**Affiliations:** PolicyLab at Children’s Hospital of Philadelphia, 2716 South Street, Philadelphia, PA 19146, USA; wangx10@email.chop.edu (X.W.); whittakerj@email.chop.edu (J.W.); kellomk@email.chop.edu (K.K.); garcias1@email.chop.edu (S.G.); marshalldb@email.chop.edu (D.M.); dechertte@email.chop.edu (T.D.)

**Keywords:** social determinants of health, maternal and child health needs assessment, child friendly communities, planning for maternal and child health

## Abstract

Environmental and community context earliest in the life course have a profound effect on life-long health outcomes. Yet, standard needs assessments for maternal and child health (MCH) programs often overlook the full range of influences affecting health in-utero and early childhood. To address this, we developed a methodology for assessing community risk in MCH based on six domains integrating 66 indicators across community, environment, socioeconomic indicators, and MCH outcomes. We pilot this methodology in Pennsylvania, and share examples of how local governments, planners, and public health officials across the geographic spectrum can integrate this data into community planning for improved maternal and child health.

## 1. Introduction

### 1.1. Maternal and Child Health in the US

Despite the US being an industrialized country with advanced medical technologies, maternal and child health (MCH) outcomes in the US lag behind other developed countries. While 157 of 183 countries reported decreases in maternal mortality in the last decade, the maternal mortality rate in the United States more than doubled between 2000 and 2014 and the country has the second highest maternal mortality rate among the thirty-one members of the Organization for Economic Cooperation and Development [1,2]. Moreover, the US infant mortality rate of 6.1 infant deaths per 1000 live births shares equally poor rankings and has also recently reversed course on progress made [3]. Vast geographic, racial, and ethnic disparities underlie these and nearly all MCH outcomes in the US. The preventable nature of most mortality in maternal and infant populations renders these disparities indefensible and requires urgent action of multidisciplinary stakeholders [4].

### 1.2. Social Determinates of MCH

Improving MCH relies on a deep understanding of the contributors to disparities in health outcomes. Health professionals increasingly recognize that health outcomes are profoundly shaped not just by biological factors but also by a range of other factors collectively categorized as social determinants of health (SDOH). According to the World Health Organization (WHO), SDOH are “the conditions in which people are born, grow, live, work and age” [5]. This constellation of environmental, political, economic, social and structural factors contribute to 60 percent of preventable mortality [6,7]. SDOH have significant relevance to MCH. For example, increasingly, research has pointed to neighborhood and other geographic boundaries as important determinants of perinatal and infant health. Adverse birth outcomes have been found to be associated with neighborhood-level contextual factors, including rates of poverty, rent burden, residential segregation, educational and income attainment, and violent crime [8,9,10,11,12,13].

Social determinants affect all individuals to varying degrees, however there are populations of mothers disproportionally disadvantaged by these factors—most notably, Black, Hispanic and American Indian mothers [14]. In the United States, geographic, racial, and ethnic disparities, present across most MCH outcomes, are rooted in pervasive structural racism. Structural racism is defined as a system where public policies, institutional practices, and cultural representations work to reinforce and perpetuate racial inequity [15]. Structural racism in healthcare and social service delivery contributes to inequitable experiences in access to and quality of care. In maternal health, racial disparities have been identified even in access to pain management during childbirth [16]. The pervasive toll of racism contributes to higher risks for a range of medical conditions among Black mothers, such as pregnancy-related high blood pressure and mental health conditions that threaten their lives and their infants’ lives [16,17]. Geographic disparities in maternal and infant health outcomes have also intensified over the past decade, with an expeditious decline following the epidemic of maternity ward closures in rural communities [18,19]. These closures have been more dramatic in counties with a higher percentage of Black women [20].

### 1.3. Planning to Improve Maternal and Child Health

City and regional planning is a distributive system influencing SDOH and patterns of health across a community, including maternal and child health. Planners’ stated goals are to ‘maximize the *health*, safety, and economic well-being of all people living in communities’ [21]. Planners help create a broad vision for the future of communities, integrating the diverse building blocks—transportation, housing, infrastructure, etc.—into a cohesive community plan through policy tools such as local laws, tax exemptions, and public financing. However, planners have been reticent to utilize these tools to intervene in social determinants of maternal health *to specifically plan for environments supportive of pregnant women, mothers, and infants*. Inadequacies in planning for women and families have been highlighted in critiques of suburbia as an isolating urban form, occupied by, but not designed for, the daytime activities of women and children [22]. Housing structure, too, has not been built for the needs of women and families, particularly working mothers [23]. Outside of the suburbs (and increasingly within the suburbs), acknowledgement of the feminization of poverty—that women with children constitute a large majority of low-income families—has not led to supportive planning practice for families [24,25]. Few comprehensive community plans include specific consideration of children [26]. Today, planning has not openly grappled with the need for increasingly popular social policies, like universal childcare or family leave, and municipal governments have often failed to provide adequate services, like public transportation, to caregivers in the locations where they can be used [23,25].

There are few contemporary examples within the planning discipline of *plans* that utilize maternal and early child health indicators such as infant and child mortality, childhood blood lead levels, racial disparities in low birth weights, and availability of childcare providers (though, the emphasis on childcare as infrastructure continues to gain momentum) [27]. The American Planning Association’s (APA) *Healthy Planning*, a gold standard guide for assisting practicing planners in engaging in healthier community planning, does not mention maternal health. Children are incorporated only when the health outcome of concern is childhood obesity, and access to greenspace and physical activity are the solution. Likewise, the APA’s *Healthy Plan Making*, designed to improve comprehensive planning for health, gives similarly little attention to maternal and child health. Though planners may not be focusing explicitly on maternal and child health as part of their professional duties, their desire to do so is strong: a survey of the profession indicates that 98% of practicing planners believe they have a role to play in planning family-friendly communities through addressing housing, design, transportation, and schools. Similarly, MCH researchers and practitioners have yet to fully realize the discipline’s voice on local planning and have been slow to operationalize robust interdisciplinary partnerships to assess and plan to reduce inequities in MCH that are rooted in structural disparities.

As attention towards both the social determinants of health and planning for mother-and-child-friendly communities evolves, we developed a set of indices that function within a larger comprehensive needs assessment to understand the MCH landscape in local communities. The MCH need indices offer a systematized way to identify the geographic disparities in health influenced by deep-rooted structural issues in the communities. Particular attention was given to examining the broader range of determinants of maternal and child health, including community, environment, and place-based indicators. We piloted these need indices in Pennsylvania, as part of a larger federally mandated public health program assessment. The data and process of these indices is closely aligned with the goals of the comprehensive planning process, providing an opportunity for incorporating this maternal and child health focus into plan-making. Furthermore, the county-level scale of the indices aligns with county-level planning more common in rural communities, making the indices useful for rural planners with few other resources for engaging in place-based improvements in maternal and child health. In this paper, we describe the development and results of the index, and provide guideposts for how these measurements of social determinants of maternal and child health could be better incorporated into traditional planning processes.

## 2. Methods

### 2.1. Selection of Indicators for MCH Needs

In an effort to provide a comprehensive view of the landscape of family and community well-being and the structural determinants of MCH, we selected 66 indicators (metrics) within 6 domains to derive the MCH need indices. The six domains include: (a) perinatal, infant, and child outcomes (11 indicators), (b) socioeconomic status (11 indicators), (c) substance use (14 indicators), (d) child safety and maltreatment (9 indicators), (e) environment and community (16 indicators), and (f) childcare (5 indicators). These indicators were informed by life course and ecosocial theories [28,29]. Life course theory asserts that the development and maintenance of health is influenced by biological, environmental, behavioral, and social factors across the lifespan and that the impacts of these influences on an individual are cumulative [30]. Ecosocial theory describes a ‘social production of disease’ rooted in the interplay between biological processes and social conditions. Ecosocial theory is a framework for identifying and understanding health inequities in populations and elevates the role of societal systems including historical, political, economic, and social systems [31]. In accordance with these theories, the indicators included in this index feature resource-focused measures that reflect the economic, structural, and historical contexts of communities alongside outcome-focused indicators that are direct measures of health status. See Table 1 for the full list of indicators.

### 2.2. Data Source and Definitions for Indicators

Most indicators were derived from raw data for Pennsylvania counties accessed from publicly available administrative data (e.g., National Vital Statistics System) and national or regional survey data (e.g., National Survey of Drug Use and Health). We also derived county-level estimates for indicators using data which are not available in public aggregate data but are administrative data processed by each state, including birth certificate records and medical billing claims data. These data sources were important for creating indicators with a high degree of specificity to the MCH population. The indicators developed using these state administrative data files included: maternal depression, well-baby visits, young child well-child visit, postpartum high-risk opioid use, pregnancy and postpartum substance use disorder, abuse against pregnant and postpartum women, infant non-superficial injury, and young child non-superficial injury.

In the primary domain need analyses, raw data was standardized to the county level. Data manipulations were performed as needed to standardize metrics for comparisons across counties. For example, when the raw data only contained absolute numbers (e.g., number of infant deaths in each county per year), appropriate denominators (e.g., number of live births in each county per year) were added to create rates (e.g., deaths per 1000 live births) to account for the difference in population size when comparing county estimates. See Table 1 for indicator definitions. See Supplementary 1 for the data year of each indicator and the statewide statistics of the derived indicators in 67 Pennsylvania counties. See Supplementary 2 for a detailed description of how the indicators were obtained and measured.

We chose counties as units in primary analyses with the following justification: (1) this study is part of a larger federally mandated county-based needs assessment overseen by the Health Resources and Services Administration (HRSA) [32], (2) in accordance with federal funding streams for maternal and child health programming, state public health officials most frequently use counties as geographic units when allocating resources for community-based maternal and child public health services, and (3) the approach of using county as a primary geographic unit for evaluating community needs is consistent with existing widely used population health needs assessments and ranking systems, such as the County Health Rankings and Roadmaps (CHR&R) [33]. By ranking the health of nearly every county in the US, CHR&R compiles county-level measures from a variety of national data sources and ranks counties within each state on a selected set of health outcomes and health-related factors. The need indices in our study focused specifically on maternal and child populations and further refined existing county-level indicator systems to support maternal and child health. Although data at more refined geographies can provide important information on meaningful within-county heterogeneity, including racial and economic health disparities, sub-county-level data are often less accurate or accessible than county-level data (especially for rural counties). Qualitative methodologies, including purposively sampled surveys or focus groups, are appropriate mechanisms for identifying disparities that may be masked in county-level data review.

### 2.3. Need Score on Each Indicator

Quartiles were used to define counties with elevated need. This quartile-based method was chosen for the following reasons. First, as a ranking-based method, it aligns well with the Maternal and Child Health Bureau’s (MCHB) guidance for defining at-risk communities—“At-risk communities are those for which indicators, in comparison to statewide indicators, demonstrated that the community was at greater risk than the state as a whole” [32]. Second, it accounts for the non-normal distribution of most county-level estimates and, therefore, performs better than Z-score-based methods that assume normal distribution of county estimates. Third, as a ranking-based approach, it is stable when the absolute county estimates change significantly over the years as new data are updated, while the relative level of need between counties remains generally stable over time.

In this quartile-based method, if a county’s estimate on any specific indicator is within the top 25% of state distribution of the indicator estimates, the county was defined as having elevated need for the indicator (indicator need score = 1; otherwise = 0). For a small number of indicators (e.g., percent of regulated childcare providers meeting high-quality standards, number of substance treatment facilities per 100,000 residents), for which it was assumed that higher estimates indicate better resources and, therefore, better population health outcomes, a county was defined as elevated need if its estimate is within the lowest 25% of state distribution of that indicator.

### 2.4. A Composite Need Index on Each Domain

For each of the six domains, a county’s domain composite need score is calculated as the weighted average of the need scores of the indicators within that domain. To account for the heterogeneity between indicators in their data quality and proximity of influence on maternal and child health, a weighting scheme was used. The following metrics were considered in the weight scheme: whether or not the indicator was referenced as a requirement in official federal guidance for needs assessment protocols (1 = yes, 0 = no), proximity of impact on maternal and child health (scale of 1 to 3; score of 3 represents that literature suggests the indicator, such as preterm birth rate, to be a proximal indicator of MCH; score of 1 represents that the indicator, such as libraries in a community, is a distal influencing factor for MCH), data recency (1 = data after 2016; 0 = data before 2016), strength of data collection methodology (1 = low quality; 2 = high quality), and specificity of the population of reference in the indicator (i.e., how representative is the indicator to the home visiting target population which are low-income pregnant women or families with young children, 1 = age or pregnancy status is reflected in the indicator’s denominator or numerator; 0 = not). A weight for each indicator was calculated by adding the above metrics. See Supplementary 3 for a description of how the weight was calculated for each indicator.

Domain need score was then categorized into a need index using a quartile-based method: A county was categorized as having “elevated need” in a domain if the county’s composite need score ranked within the top 25% of all Pennsylvania counties, as having “low need” if within the bottom 25%, and the rest of the counties as “moderate need”.

### 2.5. Sub-County Analyses

In a state as large and diverse as Pennsylvania, a county-level analysis may not be sufficient for revealing local-level variation. To provide a closer view of local need and to avoid masking underlying intra-county disparities, we included a sub-county analysis of a set of indicators on which zip-code-level estimates are available. This analysis is especially useful for counties with high population density or in counties with significant income or geographic variation. Twelve counties were chosen for zip-code-level assessments. We used an empirical approach to identify counties with significant regional heterogeneity. The approach used the indicator “Poverty Rate for Children Under 5”, and calculated the percentage of zip codes within each county that fall into the “low” and “elevated” need categories, and selected a list of counties with more than 20% of zip codes in the elevated need category and more than 20% of zip codes in the low need category. Counties meeting both thresholds were included. Zip code-level need indices were generated using the same method as county-level need indices by comparing zip code estimates to the state quartiles, as described above. Results are presented visually as maps.

## 3. Results

### 3.1. Indicator-Level Results

Table 2 and Table 3 represent the indicators’ need scores and a composite domain need index for two domains as examples. The Perinatal, Infant, and Child Outcomes Domain (Table 2) is used as an example for an outcome-focused domain in which most indicators are direct health outcome measurements (e.g., preterm birth rate). The Community and Environment Domain (Table 3) is used as an example for a resource-focused domain in which most indicators reflect the economic and environmental context that shape resource availability with downstream effects on health. In each table, we present the results for six counties as examples—two counties in each of three categories of a domain composite need index (elevated, moderate, or low need). In Table 2, the overall need for the whole Perinatal, Infant, and Child Outcomes Domain was summarized into domain composite need score and need index. The need score was determined as a weighted sum of a county’s need scores on all indicators within the domain. In Table 3, we also calculated composite need score and index by summarizing indicators within the Community and Environment Domain.

The results presented in Table 2 and Table 3 show the diverse landscape of health behaviors, health and social services access, and environmental exposures present across counties in a single US state. Table 2 shows that there was large heterogeneity in breastfeeding initiation rates across Pennsylvania, with some counties exhibiting near universal breastfeeding at hospital discharge and other counties with near 50% of live births not breastfed at discharge. Related to recommended preventive care utilization, early prenatal care initiation was common in more counties than was on-time well-child visits for infants and young children. In 39 of 67 counties, three out of every four births were to mothers who initiated prenatal care in the first trimester. Conversely, only 22 counties met or exceeded the American Academy of Pediatrics (AAP) recommendation of six or more well-baby visits in the first year of life, and children aged 1–5 in 11 counties had, on average, less than 1 well-child visit per age year.

Table 3 shows that there was also substantial heterogeneity across indicators in the Community and Environment Domain. While some counties had as many as half of the census tracts designated low income and low access, defined by the United States Department of Agriculture (USDA) as 30% or more of residents live over 10 miles from a food store, other counties had no low-income and low-access census tracts. Similar variation was observed in libraries per capita. In nine counties, more than 1 in 10 children experienced elevated blood lead levels. Moreover, the availability of healthcare resources varied widely across the state. The number of community health centers per 100,000 residents had a wide range across the 67 counties (from 0 to 45). While most counties had more than three primary care physicians per 1000 residents, 37 out of the 67 counties had no pediatric dentists.

### 3.2. Overall Domain Results

**Figure 1:**
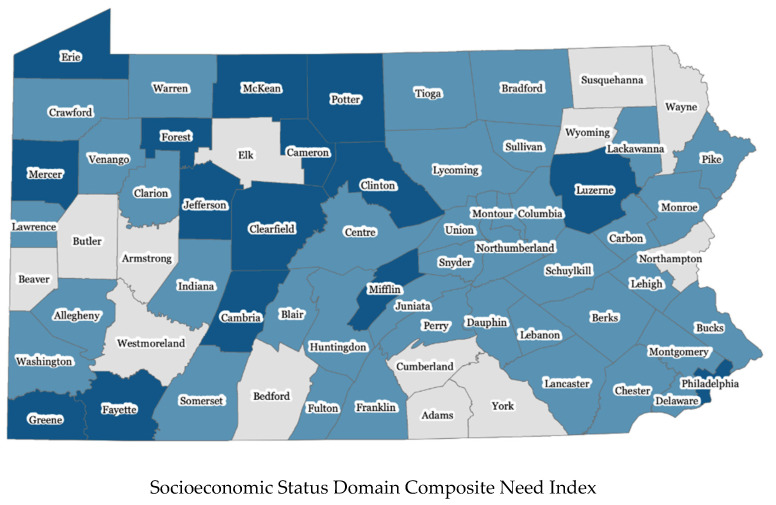

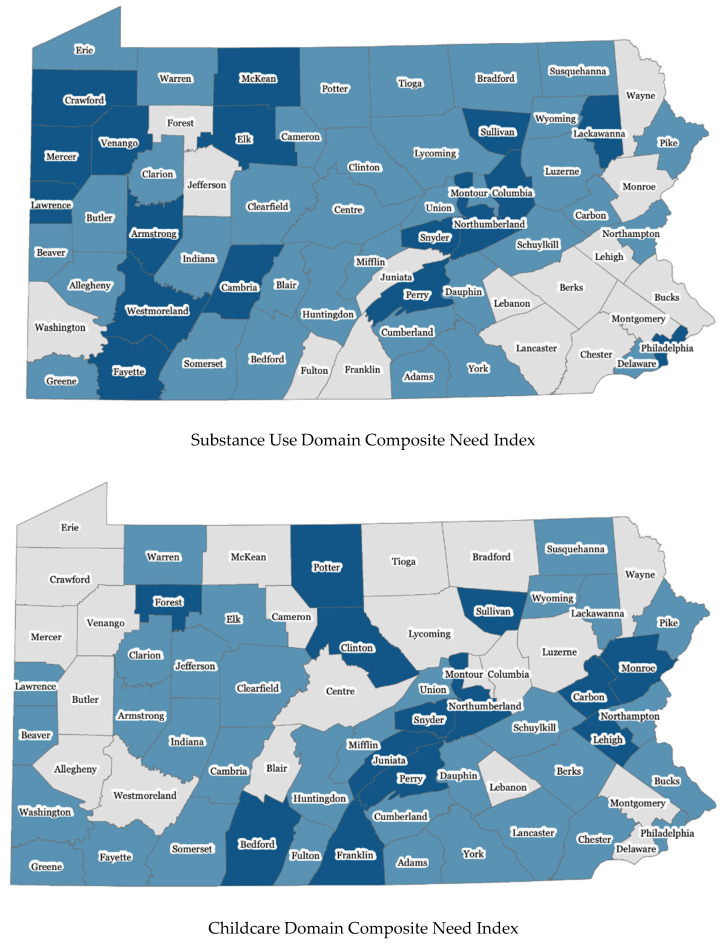

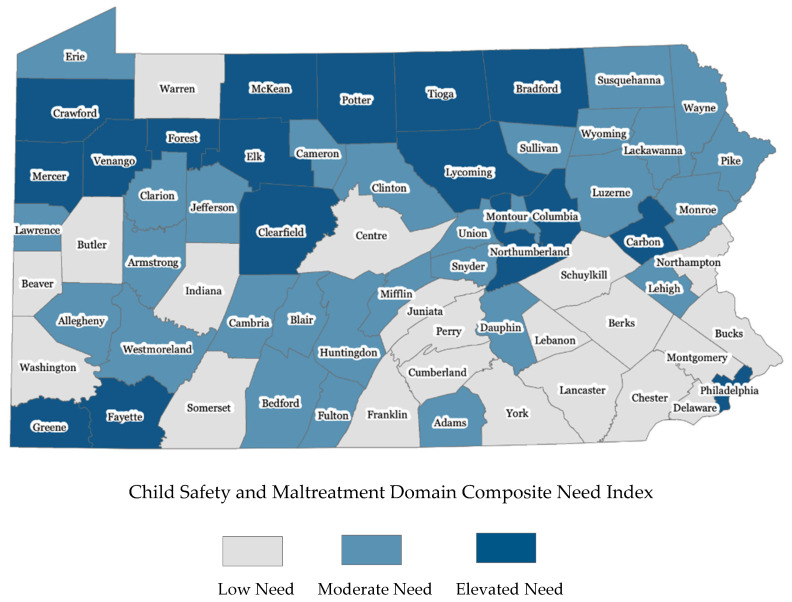
Domain composite need indices, in 67 counties in Pennsylvania.

In addition to the above two domains, we used the same approach and calculated the composite domain need indices for the other four domains. Figure 1 presents the composite need indices of each county on the Socioeconomic Status Domain, Substance Use Domain, Childcare Domain, and Child Safety and Maltreatment Domain. Of the 67 counties in Pennsylvania, 23 counties did not meet the elevated need threshold for any of the six domains, 44 counties reached elevated need status in at least one domain, and 15 counties met elevated need thresholds in three or more domains. Concentrations of need were dispersed across rural, urban, and suburban counties in the state.

Table 4 presents the correlation between need index on Perinatal, Infant, and Child Outcomes Domain and the need indices on the other 5 domains. Supplementary 4 further presents the correlations between the 6 domains. Overall, patterns of correlated risks were not strongly identifiable. Each county’s profile of risk and strength has unique components. Among the 16 counties at elevated need for the Perinatal, Infant, and Child Outcomes Domain, 7 (43%) of them were also at elevated need on the Socioeconomic Status Domain and 8 (50%) of them were also at elevated need on the Child Safety and Maltreatment Domain.

### 3.3. Sub-County Results

**Figure 2:**
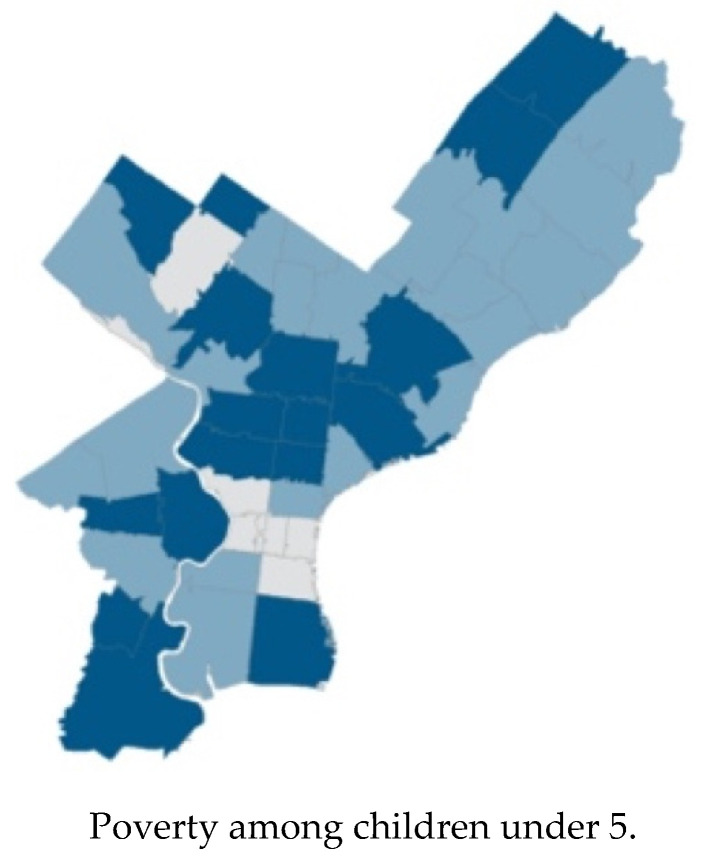

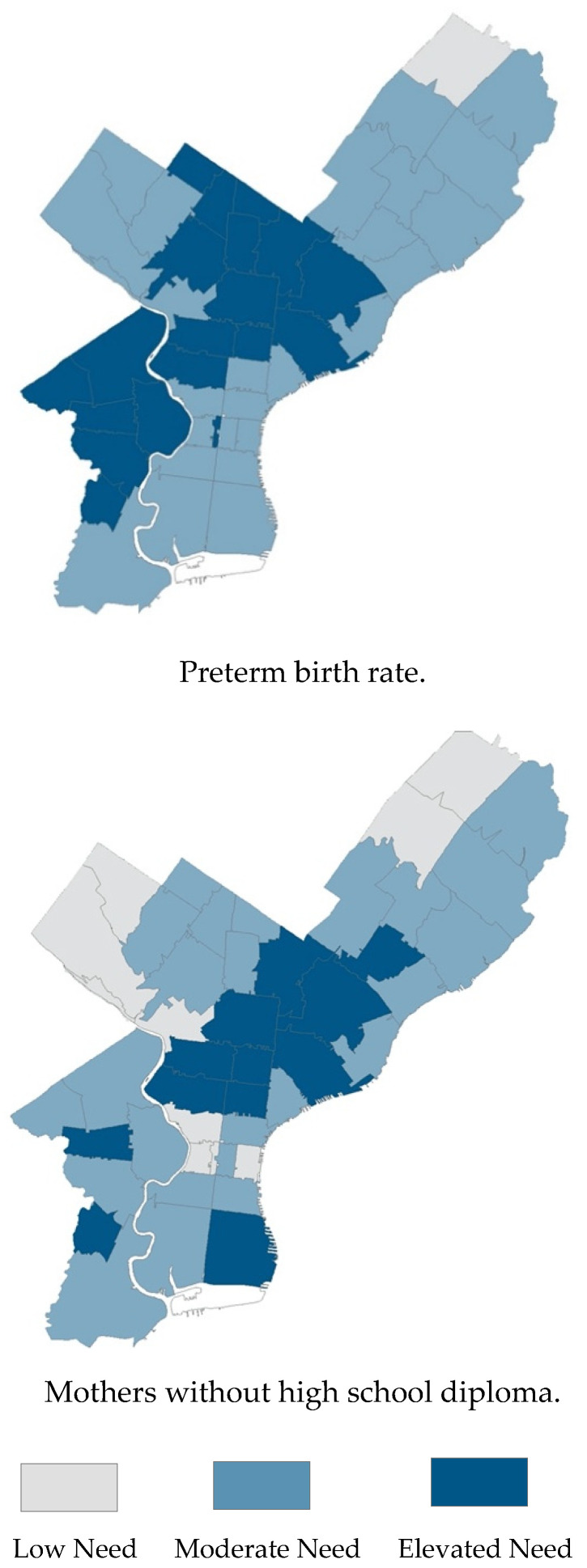
Zip-code level need indices, using Philadelphia County, PA, as an example.

Sub-county results for Philadelphia County are included in this manuscript. Figure 2 presents the need indices of Philadelphia County for three indicators at the zip code level. Philadelphia County displays significant heterogeneity across zip codes, on poverty, preterm birth, and high school completion of mothers. In general, neighborhoods near the geographic center of Philadelphia experience higher needs than the rest of the county.

## 4. Discussion

### 4.1. Incorporating Social Determinants in Evaluating Maternal and Child Health Needs

Needs assessments are a critical public health and planning tool, but the process for creating a comprehensive product is challenging. Previous maternal and child health needs assessments have sometimes failed to achieve maximum community benefits because measurement metrics have been limited to proximal health outcomes, overlooking the full range of factors, including factors within the purview of planning, that influence maternal and child health [34]. This approach is reflected in the healthcare system too. Efforts to improve maternal outcomes have often focused on informing or encouraging individuals to modify behaviors, without taking into account their physical and social environments. This method has failed to reduce health inequalities and led to disappointing patterns such as an increase in preterm births [35,36].

In this needs assessment, we incorporated SDOH through a range of environmental, political, economic, social and structural factors linked to maternal and child health outcomes. In particular, we incorporated measures for which there is evidence of interventions to reduce social inequalities in health: stress, early life, social exclusion, work, employment, social support, food, and transportation [37]. Although there is no simple solution to the complex problem of health disparities, promising and knowledge-based directions for action have been explored, including economic development initiatives targeting and engaging disadvantaged communities, community-focused initiatives that can lead to healthier communities, federal grant programs targeted to provide comprehensive family planning services and preventive health services [38], and home visitation programs giving pregnant women and families, particularly those considered at-risk, necessary resources and skills to be physically and emotionally healthy [39]. For example, the Best Babies Zone (BBZ) Initiative is a collaborative, place-based effort to mobilize four sectors—healthcare, early care and education, economic development, and community systems—to address the social determinants of health and improve birth outcomes [40]. BBZ works holistically to improve living conditions and opportunities for families by aligning resources, building community leadership, and transforming educational opportunities, economic development, and community systems in concentrated neighborhoods.

### 4.2. Incorporating Maternal and Child Health Needs with Planning

A maternal and child health needs assessment geared towards the social determinates of health has much to offer to planners designing family-friendly communities. Despite the planners’ stated goal of creating healthy communities and multiple tools planners have to address social determinants of health, planning has not always distributed public goods well for children and parents. Families with children are sometimes viewed (by planners) as a regional drain, not generating sufficient tax revenue to offset the cost of the community services they demand [26]. In 2008, the American Planning Association surveyed their membership with questions pertaining to planning family-friendly cities. Results of the survey point to little awareness of services specific to families with young children; for example, slightly over half (57%) of participants explicitly referenced meeting families’ needs via their comprehensive plan, and only 43% of respondents were aware of whether their community had an adequate supply of childcare and only 5% had a childcare plan [26]. While scholarship connecting the pathways between structural and social determinants and health outcomes has blossomed, planners may not be operationalizing these pathways to plan for environments supportive of maternal and child health.

One reason for the disconnect between planning practice and efforts to improve maternal and child health is the (understandable) lack of knowledge within the planning profession of what, within their professional purview, influences maternal and child health. To address this gap in planning knowledge, we developed need indices that can be easily utilized by planners, local governments, and other stakeholders to evaluate and understand maternal and child health metrics in their region. Armed with this indicator data, planners can then visualize which action items are within their scope of influence.

Our multidisciplinary team developed several facets of the assessment that we feel assist in making this a planning-friendly needs assessment. First, we developed the indices at different geographic levels. While county-level need indices are helpful for planners who work for the state or county level, zip-code-level need indices may better facilitate local planners working at the municipal or community level. Second, we utilized our skills as MCH researchers and practitioners to gather existing scientific evidence on how multiple social determinants interactively affect MCH outcomes and input them into the development of the need indices. With this completed, planners can understand and measure how their professional endeavors can improve MCH without having to disentangle the complex connections between a large number of MCH-related metrics. In addition to individual MCH need scores on each metric, we simplified the indicators into a composite need score for a whole domain. These composite need scores provide planners with synthesized and straightforward knowledge on the overall status of maternal and child health outcomes and related factors. For example, if a county scored low in the community environment domain, this is a sign that planners may have an outsized role to play in improving MCH outcomes in that community. Third, while most previous MCH needs assessments focus on proximal health outcomes when selecting metrics to be included in the need indices, we emphasized resource-based indicators that reflect the economic and social contexts of communities where families live. While healthcare providers and public health workers directly intervene on proximal health outcomes, planners work in the public sector and use broad policy tools such as local laws, plans, standards, public financing tools, and other policies to manipulate social and physical environments that contribute to downstream health effects. With the indicators in this needs assessment, planners can act on the resource-based indicators and intervene in the pathways from which social determinants affect health outcomes. For example, planners may help determine the location of new affordable housing construction, bus line, or grocery store, which will in turn affect the resources available to individuals and their health-related behaviors. Fourth, we used publicly available data for the majority of the indicators included in the need indices. Planners will be able to access the data from public sources without any cost and to update or filter the data to fit their customized need.

Addressing the determinants of MCH requires a multi-sector coalition. The need indices presented in this work support efforts to identify and prioritize communities for strategic investment and programmatic supports across a range of domains that impact health. The strong and growing empirical evidence of the role of structural community-level determinants of health is a call to action for disciplines who may be uniquely positioned to innovate and remedy longstanding health disparities operating above the influence of individual locus of control. While planners are well positioned, unpacking the factors contributing to a particular community need and designing and implementing programming to improve it will be most effective when undertaken in partnership with representation from public health, health, and government systems. The need indices can support a wide variety of policymakers in comparing MCH county-level metrics across the state and prioritize communities for funding. Program administrators, service providers, local policymakers, and advocacy groups can also use these local MCH need indices to inform tailored programmatic and policy responses across multiple public sectors that benefit children and families.

Our study has several limitations. First, there is no precedent for assigning weights on a large number of MCH and SDOH indicators for creating composite need indices of local communities. This study contributes a novel approach that calculates composite need indices as a weighted average of indicators. The weighting scheme was informed by MCH literature and is reflective of both data quality and content. The scheme was designed through focus group discussion among a multi-disciplinary team in the fields of maternal and child health, epidemiology, regional planning, and state public health officials. Second, we piloted the need indices in Pennsylvania and our results may not be generalizable to other geographic areas. However, the majority of our indicators were derived using national data sources, which make them readily applicable to other US areas.

## 5. Conclusions

An ever-growing body of evidence continues to stress the importance of addressing health outcomes through interdisciplinary approaches beyond the healthcare system, addressing racism, class discrimination, and unsupportive policy environments; for example, a 2020 *Pediatrics* article demonstrates the effect of government expenditures in housing, parks and recreation, public health, solid waste management, and other non-healthcare expenditures on improving infant mortality [41]. The emphasis on social and structural determinants of health—efforts required outside of the hospital and doctor’s office—offers a clear invitation for fields like planning to utilize their skillset for improving community health. From the planning perspective, this invitation echoes the earliest motivations of the discipline, a field historically concerned with reducing human suffering from rapid urbanization, crowded living conditions, polluted water, lack of sanitation infrastructure, and widespread disease [42]. Planners can and must revisit their earliest motivations and join in multi-disciplinary partnerships to address preventable maternal and infant mortality. We hope this contribution provides one such example for doing so. By disentangling the complexities of the MCH evidence base and tying it to resource-based indicators, we add a specific maternal focus to the literature and practice on planning family-friendly communities.

## Figures and Tables

**Table 1:** Indicators included in the Need Indices: Metrics on Maternal and Children Health Outcomes and Structural Determinants.

Indicator Name	Indicator Definition
Perinatal and Neonatal Outcomes Domain	
Late prenatal care	Percent of births to mothers who did not initiate prenatal care in the first trimester
Preterm birth	Percent of live births < 37 completed gestational weeks
Low birth weight	Percent of live births < 2500 g at birth
NICU admission	Percent of live births admitted to a neonatal intensive care unit (NICU)
Late/no breastfeeding initiation	Percent of live births who were NOT breastfed at hospital discharge
Infant mortality	Infant deaths per 1000 live births
Child mortality	Deaths of children under 5 years old per 1000 residents under 5
Maternal depression	Prevalence of diagnosed depression in the 2016 calendar year among Medicaid-enrolled women who were pregnant or gave birth during 2014–2016
Well-baby visits	Median number of well-child visits among Medicaid-enrolled children aged less than 1 year
Young child well-child visit	Median number of well-child visits among Medicaid-enrolled children aged 1–5 years
Racial disparity in low birth weight	Ratio of low-birth-weight rate in births born to Black mothers to that in births born to white mothers
Substance Use Domain	
Postpartum high-risk opioid use	Rate of mothers filling ≥ 2 opioid prescriptions in the 2017 calendar year among Medicaid-enrolled mothers who delivered live births during 2015–2016
Substance treatment facilities	Number of drug and alcohol treatment facilities per 100,000 residents
Mental health treatment facilities	Number of mental health treatment facilities per 100,000 residents
Buprenorphine physicians	Number of Buprenorphine treatment practitioners per 100,000 residents
Impaired drivers	Number of vehicle crashes involving impaired driver per 100,000 residents
Overdose deaths	Rate of overdose deaths per 100,000 people aged 15–64 years
Opioid overdose hospitalizations	Rate of hospitalization for opioid overdose per 100,000 residents
Neonatal abstinence syndrome	Rate of neonatal abstinence syndrome per 1000 newborn stays
Pregnancy and postpartum substance use disorder	Rate of diagnosed substance use disorder in the 2016 calendar year among Medicaid-enrolled mothers who were pregnant or delivered live births during 2014–2016
Alcohol use disorder	Prevalence rate of Alcohol Use Disorder among individuals aged 12 and older
Marijuana use	Prevalence rate of marijuana use in past month among individuals aged 12 and older
Cocaine use	Prevalence rate of cocaine use in the past year among individuals aged 12 and older
Heroin use	Prevalence rate of heroin use in the past year among 12 and older
Maternal smoking during pregnancy	Rate of births to mothers who used tobacco during pregnancy per 100 live births
Socioeconomic Status Domain	
Poverty	Percent of population living below 100% Federal Poverty Level (FPL)
Child poverty	Percent of children under age 5 living in poverty
Income inequality	Gini Coefficient 5-year estimate or 1-year estimate
Unemployment	Unemployed percent of the civilian labor force
Teens Not in School	Percent of 16–19-year-olds not enrolled in school and with no high school diploma
Teen births	Number of births per 1000 female population ages 15–19
Mothers without high school diploma	Percent of births to mothers whose educational attainment is below high school
Public assistance	Percent of households with children under 18 years who have received Supplemental Security Income (SSI), Cash Assist, or Supplemental Nutrition Assistance Program (SNAP) in the past 12 months
Renters who are cost burdened	Percent of renters who are cost burdened by rent
WIC redemptions	Per capita dollar amount of SNAP for Women, Infant, and Children (WIC) redemptions
Child food insecurity	Percent of children living in households that experienced food insecurity at some point in 2017
Child Safety and Maltreatment Domain	
Child Maltreatment	Number of children with substantiated reports of child abuse per 1000 children under 18 years old
Substantiated young child abuse and neglect	Number of substantiated child abuse and neglect per 1000 children aged 0–4
Abuse against pregnant and postpartum women	Rate of diagnosed abuse in the 2016 calendar year among Medicaid-enrolled pregnant women or women who gave live birth during 2014–2016
Domestic violence-related deaths among women of childbearing age	Number of domestic violence-related deaths per 1000 female aged 15–50 years
Protection from abuse order	Number of judge-grated protection from abuse order per 1000 residents
Infant non-superficial injury	Prevalence of children having non-superficial injury during the first year of life per 1000 Medicaid-enrolled children
Young child non-superficial injury	Prevalence of children having non-superficial injury during the first 5 years of life per 1000 Medicaid-enrolled children
Child welfare in-home services	Percent of children under 18 receiving child welfare in-home services in Fiscal Year (FY) 2017–2018
Substance Use Need	Composite need score of a set of substance use disorder-related indicators
Community Environment Domain	
SNAP-authorized stores	Number of SNAP authorized stores per 1000 families
WIC-authorized stores	Number of WIC authorized stores per 1000 families with children under 6
Low-income and low-access census tracts	Percent of census tract with low income and low access
Hospitals	Number of hospital beds per 1000 residents
Community Health Centers	Number of community Health Centers, Federally Qualified Health Centers (FQHCs), and look alikes per 100,000 residents
Primary care physicians	Number of primary care physicians per 1000 residents
Pediatric Dentists	Number of Active Clinical Pediatric Dentists per 1000 children under age 18
Crimes	Number of reported crimes per 1000 residents
Juvenile arrests	Number of crime arrests ages 0–17 per 100 juveniles aged 0–17
Environmental quality	Average index score of potential exposure to harmful toxins
Libraries	Number of libraries per 100,000 residents
Public Transit in Urban Counties	Public transit performance score in 6 urban counties (Delaware, Chester, Montgomery, Bucks, Philadelphia, and Allegheny)
Car Ownership in Rural Counties	Percent of census tracts with low car ownership in 61 rural counties
Children Blood Lead Level (BLL)	Percent of children with confirmed BLLs ≥ 5 µg/dL
Residential Segregation	Index of dissimilarity, where higher values indicate greater residential segregation between Black and White county residents
Childcare Domain	
Regulated Childcare	Number of regulated childcare providers per 100 children residents under 3 years old
High-quality Childcare	Percent of regulated childcare providers meeting high-quality standards
Subsidized Childcare	Percent of children 0–5 eligible for Child Care Works (CCW) who were served by CCW
Publicly Funded Pre-K	Percent of children aged 3–4 below 300% poverty with access to publicly funded, high-quality pre-k
Quality of Subsidized Childcare	Percent of children aged 0–5 receiving subsidized childcare in Keystone STARS (Standards, Training/Professional Development, Assistance, Resources, and Supports) program level 3 or 4 facilities

**Table 2:** Indicator need indices and a composite need index for the domain of perinatal and neonatal outcomes, in 6 example Pennsylvania counties.

	Indicator Estimates and Need Scores																						Domain Composite Need Index	
Indicator Name ^1^:	Late Prenatal Care		Preterm Birth		Low Birth Weight		NICU Admission		Late/No Breastfeeding Initiation		Infant Mortality		Child Mortality		Maternal Depression		Well-Baby Visits		Young Child Well-Child Visit		Racial Disparity in Low Birth Weight			
Indicator Weight:	7		8		8		6		6		7		7		7		7		6		7		Composite Need Score ^3^	Composite Need Index ^4^
	Raw Value	Need Score ^2^	Raw Value	Need Score ^2^	Raw Value	Need Score ^2^	Raw Value	Need Score ^2^	Raw Value	Need Score ^2^	Raw Value	Need Score ^2^	Raw Value	Need Score ^2^	Raw Value	Need Score ^2^	Raw Value	Need Score ^2^	Raw Value	Need Score ^2^	Raw Value	Need Score ^2^		
Example counties:																								
Mercer	31.6	1	9.5	0	7.2	0	6.2	0	27.6	0	7.8	1	2.2	1	15.5	1	3	1	0	1	2.5	1	0.63	Elevated
Dauphin	31.2	1	10.1	1	9.1	1	9.9	1	15.2	0	7.8	1	1.9	1	7.7	0	5	0	1	0	1.9	0	0.57	Elevated
Allegheny	12.4	0	9.3	0	7.8	0	11.0	1	20.3	0	5.9	0	1.4	0	10.3	0	4	1	1	0	2.2	1	0.26	Moderate
Butler	17	0	8.5	0	6.1	0	8.8	0	18.2	0	2.8	0	0.5	0	11.9	0	3	1	0	1	2.3	1	0.26	Moderate
Lehigh	22.5	0	9.2	0	8.1	0	9.1	0	16.6	0	6.2	0	1.3	0	8.8	0	5	0	1	0	1.8	0	0.0	Low
Montgomery	21.3	0	8.4	0	7.4	0	8.4	0	9.2	0	4.8	0	1.1	0	5.7	0	5	0	1	0	1.9	0	0.0	Low
State-wide statistics:																								
Min	12.4		6.1		4.8		3.5		2.6		0.0		0.0		3.0		2		0		0.8		0.0	
25th Percentile	20.8		8.3		7.0		5.7		16.0		3.1		0.8		9.9		4		1		1.6		0.09	
75th Percentile	30.2		9.7		8.1		9.3		27.6		7.2		1.7		14.5		6		1		2.0		0.37	
Max	38.9		11.7		10.6		20.7		46.2		14.4		3.6		18.4		7		2		2.5		0.63	

Grey cells show the need indices we created for each county. ^1^ See Table 1 for detailed definitions, data sources, and data year of indicators. NICU: newborn intensive care unit. ^2^ Indicator need score was defined using a quartile-based method. For most indicators in this domain (Late prenatal care, Preterm birth, Low birth weight, NICU admission, Late/no breastfeeding initiation, Infant mortality, Child mortality, Maternal depression, and Racial disparity in low birth weight) for which it was assumed that higher estimates indicate worse population health outcomes, a county was defined as having elevated need for the indicator (indicator need score = 1; otherwise = 0) if the county’s estimate on the indicator is within the top 25% of state distribution. For two indicators in this domain (Well-baby visits and Young child well-child visit) for which it was assumed that higher estimates indicate better resources and better population health outcomes, a county was defined as having elevated need for the indicator (indicator need score = 1; otherwise = 0) if the county’s estimate on the indicator is within the lowest 25% of state distribution. ^3^ A county’s domain composite need score is calculated as the weighted average of the need scores of the indicators within that domain. The following metrics were considered in the weighting scheme: whether or not US Maternal and Child Health Bureau (MCHB) has referenced it as a requirement in official guidance (1 = yes, 0 = no), direct impact on maternal and child health (scale of 1 to 3; score of 3 represents that literature suggests the indicator to be a proximal indicator of maternal and child health), data recency (1 = data after 2016; 0 = data before 2016), strength of data collection methodology (1 = low quality; 2 = high quality), and specificity of the population of reference in the indicator (i.e., how representative is the indicator to the home visiting target population, 1 = age or pregnancy status is reflected in the indicator’s denominator or numerator; 0 = not). A weight for each indicator was calculated by adding the above metrics. The weight assigned on each indicator was presented as the third row of the table. ^4^ A county was categorized as having “elevated need” in this domain if the county’s composite need score ranked within the top 25% of all Pennsylvania counties, as having “low need” if within the bottom 25%, and the rest of the counties as “moderate need”.

**Table 3:** Indicator need indices and a composite need index for the domain of Community and Environment, in 6 example Pennsylvania counties.

Indicator Estimates and Need Scores																													Domain Composite Need Index	
Indicator Name ^1^	SNAP Stores		WIC-Stores		Low-income & Low-Access Census Tracts		Hospitals		Community Health Centers		Physicians		Pediatric Dentists		Crimes		Juvenile Arrests		Environment Quality		Libraries		Car Ownership		Children Blood Lead		Residential Segregation			
Indicator Weight	3		3		4		4		6		6		5		5		5		4		4		4		7		5		Composite Need Score ^3^	Composite Need Index ^4^
	Raw Value	Need Score ^2^	Raw Value	Need Score ^2^	Raw Value	Need Score ^2^	Raw Value	Need Score ^2^	Raw Value	Need Score ^2^	Raw Value	Need Score ^2^	Raw Value	Need Score ^2^	Raw Value	Need Score ^2^	Raw Value	Need Score ^2^	Raw Value	Need Score ^2^	Raw Value	Need Score ^2^	Raw Value	Need Score ^2^	Raw Value	Need Score ^2^	Raw Value	Need Score ^2^		
Example counties:																														
Monroe	4.1	1	3.2	0	3.0	0	1.4	1	0.0	1	0.4	1	0.00	1	21.6	1	1.9	1	72	0	4.2	1	24.2	0	0.01	0	34	0	0.59	Elevated
Juniata	6.2	0	1.8	1	20.0	1	0.0	1	0.0	1	0.2	1	0.00	1	8.2	0	1.1	0	90	0	4.1	1	60.0	0	0.04	0	66	1	0.57	Elevated
Northampton	5.1	1	3.4	0	7.4	0	3.5	0	0.7	0	0.8	0	0.05	0	17.0	0	1.6	0	60	1	3.3	1	42.7	0	0.11	1	44	0	0.28	Moderate
Northumberland	7.9	0	3.1	0	8.3	0	0.8	1	1.1	0	0.5	0	0.00	1	15.5	0	2.0	1	79.5	0	7.6	0	66.7	1	0.08	0	53	0	0.28	Moderate
Columbia	7.9	0	3.3	0	0.0	0	2.6	0	1.5	0	0.6	0	0.08	0	15.3	0	1.2	0	87	0	4.5	0	40.0	0	0.06	0	52	0	0.00	Low
Wayne	7.1	0	5.1	0	7.1	0	1.7	0	15.6	0	0.5	0	0.11	0	12.6	0	0.7	0	93	0	11.7	0	14.3	0	0.07	0	43	0	0.00	Low
State-wide statistics:																														
Min	2.7		1.4		0.0		0.0		0.0		0.0		0.00		7.9		0.7		20		2.2		14.3		0.01		34		0.00	
25th Percentile	5.7		2.6		1.0		1.7		0.5		0.5		0.00		12.6		1.2		71		4.3		38.7		0.04		51		0.18	
75th Percentile	8.0		4.3		13.2		3.8		4.0		0.8		0.08		17.8		1.9		92		10.4		60.0		0.08		65.5		0.35	
Max	15.8		47.6		50.0		42.5		44.5		4.5		0.27		41.5		5.6		97		29.5		100		0.29		76		0.58	

Grey cells show the need indices we created for each county. ^1^ See Table 1 for detailed definitions, data sources, and data year of indicators. SNAP: US Supplemental Nutrition Assistance Program. WIC: Special Supplemental Nutrition Program for Women, Infants, and Children. ^2^ Indicator need score was defined using a quartile-based method. For some indicators in this domain (Low-income and low-access census tracts, Crimes, Juvenile arrests, Car Ownership, Children Blood Lead, and Residential Segregation) for which it was assumed that higher estimates indicate worse population health outcomes, a county was defined as having elevated need for the indicator (indicator need score = 1; otherwise = 0) if the county’s estimate on the indicator is within the top 25% of state distribution. For the rest of the indicators in this domain (SNAP stores, WIC stores, Hospitals, Community Health Centers, Physicians, Pediatric Dentists, and Libraries) for which it was assumed that higher estimates indicate better resources and better population health outcomes, a county was defined as having elevated need for the indicator (indicator need score = 1; otherwise = 0) if the county’s estimate on the indicator is within the lowest 25% of state distribution. ^3^ A county’s domain composite need score is calculated as the weighted average of the need scores of the indicators within that domain. The following metrics were considered in the weighting scheme: whether or not MCHB has referenced it as a requirement in official guidance (1 = yes, 0 = no), direct impact on maternal and child health (scale of 1 to 3; score of 3 represents that literature suggests the indicator to be a proximal indicator of maternal and child health), data recency (1 = data after 2016; 0 = data before 2016), strength of data collection methodology (1 = low quality; 2 = high quality), and specificity of the population of reference in the indicator (i.e., how it represents the target population, 1 = age or pregnancy status is reflected in the indicator’s denominator or numerator; 0 = not). A weight for each indicator was calculated by adding the above metrics. The weight assigned on each indicator was presented as the third row of the table. ^4^ A county was categorized as having “elevated need” in this domain if the county’s composite need score ranked within the top 25% of all Pennsylvania counties, as having “low need” if within the bottom 25%, and the rest of the counties as “moderate need”.

**Table 4:** The correlation between elevated need on Perinatal, Infant, and Child Health Domain and the need indices on the other 5 domains, among 67 counties in Pennsylvania.

	Perinatal, Infant, and Child Health Domain		
	Low Need (Total 14 Counties)	Moderate Need (Total 37 Counties)	Elevated Need (Total 16 Counties)
	N of Counties (Column %)	N of Counties (Column %)	N of Counties (Column %)
**Community and Environment Domain**			
Low Need	5 (36%)	10 (27%)	2 (13%)
Moderate Need	7 (50%)	19 (51%)	7 (44%)
Elevated Need	2 (14%)	8 (22%)	7 (44%)
**Socioeconomic Status Domain**			
Low Need	4 (29%)	8 (22%)	1 (6%)
Moderate Need	10 (71%)	21 (57%)	8 (50%)
Elevated Need	0 (0%)	8 (22%)	7 (44%)
**Substance Use Domain**			
Low Need	4 (26%)	10 (27%)	3 (19%)
Moderate Need	8 (57%)	21 (57%)	6 (38%)
Elevated Need	2 (14%)	6 (16%)	7 (44%)
**Childcare Domain**			
Low Need	5 (36%)	13 (35%)	3 (19%)
Moderate Need	7 (50%)	17 (46%)	9 (56%)
Elevated Need	2 (14%)	7 (19%)	4 (25%)
**Child Safety and Maltreatment Domain**			
Low Need	6 (43%)	13 (35%)	2 (13%)
Moderate Need	5 (36%)	18 (49%)	6 (38%)
Elevated Need	3 (21%)	6 (16%)	8 (50%)

## Supplementary Materials

The following are available online at https://www.mdpi.com/1660-4601/17/24/9224/s1. Supplementary 1: Includes data year and Pennsylvania statewide summary statistics for indicators included in the maternal and child health need indices; Supplementary 2: Includes detailed description how the indicators were obtained and measured; Supplementary 3: Presents how each indicator’s total assigned weight was calculated (i.e., how each indicator scores on each of the weighting metrics); Supplementary 4: Presents the correlations between need indices of the 6 domains.
