# Supplementary material for: Integrating the Built and Social Environment into Health Assessments for Maternal and Child Health: Creating a Planning-Friendly Index
Source: Int J Environ Res Public Health. 2020 Dec 10;17(24):9224. doi: 10.3390/ijerph17249224 (PMC7763863; doi:10.3390/ijerph17249224)
Supplement: Supplementary file 1 [file ijerph-17-09224-s001.zip › supplementary 1.docx]

Supplementary 1

Includes data year and Pennsylvania statewide summary statistics for indicators included in the maternal and child health need indices.

**Table S1.** Data year and state summary statistics of indicators included in the maternal and child health need indices.

| **Indicators** | | **Definition** | **Data Year** | **Pennsylvania statewide statistics** | | |
| --- | --- | --- | --- | --- | --- | --- |
|  |  |  |  | **Min** | **Median** | **Max** |
| **Perinatal and Neonatal Outcomes Domain:** | |  |  |  |  |  |
|  | Late prenatal care | Percent of births to mothers who did not initiate prenatal care in the first trimester | 2016 | 12.4 | 23.50 | 38.9 |
|  | Preterm birth | Percent of live births <37 completed gestational weeks | 2013-2017 | 4.6 | 9.13 | 11.4 |
|  | Low birth weight | Percent of live births <2500 grams at birth | 2013-2017 | 4.8 | 7.55 | 11.0 |
|  | NICU admission | Percent of live births admitted to a neonatal intensive care unit (NICU) | 2016 | 3.5 | 7.79 | 20.7 |
|  | Late/no breastfeeding initiation | Percent of live births who were NOT breastfed at hospital discharge | 2016 | 2.6 | 22.27 | 46.2 |
|  | Infant mortality | Infant deaths per 1000 live births | 2016 | 0.0 | 5.64 | 14.4 |
|  | Child mortality | Deaths of children under 5 years old per 1000 residents under 5 | 2016 | 0.0 | 1.33 | 3.6 |
|  | Maternal depression | Prevalence of diagnosed depression in the 2016 calendar year among Medicaid-enrolled women who were pregnant or gave birth during 2014-2016 | 2016 | 3.0 | 12.05 | 18.4 |
|  | Well-baby visits | Median number of well-child visit among Medicaid-enrolled children aged less than 1 year | 2017-2018 | 2.0 | 5 | 7.0 |
|  | Young child well-child visit | Median number of well-child visit among Medicaid-enrolled children aged 1-5 years | 2017-2018 | 0.0 | 1 | 2.0 |
|  | Racial disparity in low birth weight* | Ratio of low-birth-weight rate in births born to Black mothers to that in births born to white mothers | 2014-2018 | 0.8 | 1.86 | 2.4 |
| **Substance Use Domain:** | |  |  |  |  |  |
|  | Postpartum high-risk opioid use | Rate of mothers filling >=2 opioid prescriptions in the 2017 calendar year among Medicaid-enrolled mothers who delivered live births during 2015-2016 | 2017 | 2.5 | 9.21 | 20.5 |
|  | Substance treatment facilities* | Number of drug and alcohol treatment facilities per 100,000 residents | 2018 | 0.0 | 3.31 | 21.8 |
|  | Mental health treatment facilities* | Number of mental health treatment facilities per 100,000 residents | 2018 | 0.0 | 3.74 | 17.7 |
|  | Buprenorphine physicians | Number of Buprenorphine treatment practitioner per 100,000 residents | 2018 | 0.0 | 5.39 | 38.5 |
|  | Impaired drivers | Number of vehicle crashes involving impaired driver per 100,000 residents | 2017 | 41.9 | 107.36 | 164.2 |
|  | Overdose deaths | Rate of overdose deaths per 100,000 people aged 15-64 years | 2017 | 0.0 | 29.00 | 77.0 |
|  | Opioid overdose hospitalizations | Rate of hospitalization for opioid overdose per 100,000 residents | 2016-2017 | 23.4 | 52.40 | 102.1 |
|  | Neonatal abstinence syndrome | Rate of neonatal abstinence syndrome per 1,000 newborn stays | 2016-2017 | 3.2 | 15.70 | 76.0 |
|  | Pregnancy and postpartum substance use disorder | Rate of diagnosed substance use disorder in the 2016 calendar year among Medicaid-enrolled mothers who were pregnant or delivered live births during 2014-2016 | 2016 | 2.4 | 5.35 | 15.0 |
|  | Alcohol use disorder | Prevalence rate of Alcohol Use Disorder among individuals aged 12 and older | 2014-2016 | 4.8 | 5.83 | 7.1 |
|  | Marijuana use | Prevalence rate of marijuana use in past month among individuals aged 12 and older | 2014-2016 | 5.5 | 7.02 | 13.9 |
|  | Cocaine use | Prevalence rate of Cocaine Use in the Past Year among individuals aged 12 and older | 2014-2016 | 1.1 | 1.28 | 3.3 |
|  | Heroin use | Prevalence rate of Heroin Use in the Past Year among 12 and older | 2014-2016 | 0.4 | 0.61 | 0.9 |
|  | Maternal smoking during pregnancy | Rate of births to mothers who used tobacco during pregnancy per 100 live births | 2015 | 4.3 | 16.25 | 41.5 |
| **Socioeconomic Status Domain:** | |  |  |  |  |  |
|  | Poverty* | Percent of population living below 100% Federal Poverty Level (FPL) | 2014-2018 | 6.1 | 12.66 | 24.9 |
|  | Child poverty* | Percent of children under age 5 living in poverty | 2014-2018 | 7.5 | 21.16 | 34.3 |
|  | Income inequality* | Gini Coefficient 5 year estimate or 1 year estimate | 2014-2018 | 0.4 | 0.43 | 0.5 |
|  | Unemployment* | Unemployed percent of the civilian labor force | 2019 | 3.2 | 4.7 | 6.8 |
|  | Teens Not in School | Percent of 16-19 year olds not enrolled in school and with no high school diploma | 2017 | 0.5 | 4.29 | 19.0 |
|  | Teen births | Number of births per 1,000 female population ages 15-19. | 2011-2017 | 4.0 | 22.0 | 37.0 |
|  | Mothers without high school diploma | Percent of births to mothers whose educational attainment is below high school | 2017 | 0.0 | 0.13 | 0.3 |
|  | Public assistance* | Percent of households with children under 18 years who have received SSI, Cash Assist, or SNAP in the past 12 months | 2013-2017 | 10.2 | 25.59 | 51.3 |
|  | Renters who are cost burdened* | Percent of renters who are cost burdened by rent | 2013-2017 | 23.5 | 39.91 | 57.7 |
|  | WIC redemptions* | Per capita dollar amount of WIC redemptions | 2012 | 5.6 | 14.41 | 37.1 |
|  | Child food insecurity* | Percent of children living in households that experienced food insecurity at some point in 2017 | 2017 | 12.1 | 17.9 | 24.4 |
| **Child Safety and Maltreatment Domain** | |  |  |  |  |  |
|  | Child Maltreatment | Number of Children with substantiated reports of child abuse per 1000 children under 18 years old | 2019 | 0.0 | 2.20 | 6.7 |
|  | Substantiated young child abuse and neglect | Number of substantiated child abuse and neglect per 1000 children aged 0-4 | 2016 | 0.0 | 2.27 | 8.3 |
|  | Abuse against pregnant and postpartum women | Rate of diagnosed abuse in the 2016 calendar year among Medicaid-enrolled pregnant women or women who gave live birth during 2014 - 2016 | 2016 | 0.0 | 0.00 | 0.0 |
|  | Domestic violence-related deaths among women of childbearing age | Number of domestic violence-related deaths per 1000 female aged 15-50 years | 2005-2019 | 0.0 | 0.22 | 1.5 |
|  | Protection from abuse order | Number of judge-grated protection from abuse order per 1000 residents | 2018 | 0.0 | 0.45 | 3.0 |
|  | Infant non-superficial injury | Prevalence of children having non-superficial injury during the first year of life per 1000 Medicaid-enrolled children | 2008-2014 | 0.0 | 0.01 | 0.0 |
|  | Young child non-superficial injury | Prevalence of children having non-superficial injury during the first 5 years of life per 1000 Medicaid-enrolled children | 2008-2014 | 0.1 | 0.07 | 0.1 |
|  | Child welfare in-home services | Percent of children under 18 receiving child welfare in-home services in FY 2017-2018 | 2017-2018 | 1.3 | 10.23 | 50.8 |
|  | Substance Use Need | Composite need score of a set of substance use disorder related indicators | 2014-2017 | 0.0 | 0.21 | 0.7 |
| **Community Environment Domain:** | |  |  |  |  |  |
|  | SNAP-authorized stores* | Number of SNAP authorized stores per 1000 families | 2012 | 2.7 | 7.21 | 15.8 |
|  | WIC-authorized stores* | Number of WIC authorized stores per 1000 families with children under 6 | 2012 | 1.4 | 3.29 | 47.6 |
|  | Low-income and low-access census tracts* | Percent of census tract with low income and low access | 2015 | 0.0 | 7.35 | 50.0 |
|  | Hospitals* | Number of hospital beds per 1,000 residents | 2016 | 0.0 | 2.60 | 42.5 |
|  | Community Health Centers* | Number of community Health Centers, FQHCs, and look alikes per 100,000 residents | 2018 | 0.0 | 1.44 | 44.5 |
|  | Primary care physicians | Number of primary care physicians per 1,000 residents | 2016 | 0.0 | 0.56 | 4.5 |
|  | Pediatric Dentists | Number of Active Clinical Pediatric Dentists Per 1,000 children under age 18 | 2017 | 37 counties had 0 | | |
|  | Crimes* | Number of reported crimes per 1,000 residents | 2016 | 7.9 | 15.60 | 41.5 |
|  | Juvenile arrests | Number of crime arrests ages 0-17 per 100 juveniles aged 0-17 | 2016 | 0.68 | 1.56 | 5.57 |
|  | Environmental quality* | Average index score of potential exposure to harmful toxins | 2015 | 20.0 | 83 | 97.0 |
|  | Libraries* | Number of libraries per 100 residents | 2015 | 2.2 | 5.49 | 29.5 |
|  | Public Transit in Urban Counties* | Public transit performance score in 6 urban counties (Delaware, Chester, Montgomery, Bucks, Philadelphia, and Allegheny) | 2016 | 2.4 | 4.90 | 9.0 |
|  | Car Ownership in Rural Counties | Percent of census tracts with low car ownership in 61 rural counties | 2017 | 14.3 | 50.00 | 100.0 |
|  | Children Blood Lead Level* | Percent of Children with Confirmed BLLs ≥ 5 µg/dL | 2017 | 0.0 | 0.06 | 0.3 |
|  | Residential Segregation* | Index of dissimilarity where higher values indicate greater residential segregation between Black and White county residents | 2014-2018 | 34.0 | 57.00 | 76.0 |
| **Child Care Domain:** | |  |  |  |  |  |
|  | Regulated Child Care* | Number of regulated child care providers per 100 children residents under 3 years old | 2018-2019 | 0.0 | 1.10 | 2.3 |
|  | High-quality Child Care | Percent of regulated child care providers meeting high-quality standards | 2018-2019 | 0.0 | 0.25 | 0.7 |
|  | Subsidized Child Care | Percent of children 0-5 eligible for CCW who were served by CCW | 2018-2019 | 0.0 | 0.23 | 0.7 |
|  | Publicly Funded Pre-K | Percent of children aged 3-4 below 300% poverty with access to publicly funded, high-quality pre-k | 2018-2019 | 0.1 | 0.41 | 0.8 |
|  | Quality of Subsidized Child Care | Percent of children aged 0-5 receiving subsidized child care in Keystone STARS 3 or 4 facilities | 2018-2019 | 0.0 | 0.42 | 0.8 |

* Areas where traditional planners could intervene.
