# Supplementary material for: Integrating the Built and Social Environment into Health Assessments for Maternal and Child Health: Creating a Planning-Friendly Index
Source: Int J Environ Res Public Health. 2020 Dec 10;17(24):9224. doi: 10.3390/ijerph17249224 (PMC7763863; doi:10.3390/ijerph17249224)
Supplement: Supplementary file 1 [file ijerph-17-09224-s001.zip › supplementary 2.docx]

Supplementary 2

Presents how each indicator’s total assigned weight was calculated (i.e., how each indicator scores on each of the weighting metrics).

**Table S2.** Weighting scheme for indicators included in the maternal and child health need indices.

|  | | **Metrics for Weights** | | | | | **Total weight** |
| --- | --- | --- | --- | --- | --- | --- | --- |
|  | | **Whether or not it has been require in HRSA official guideline** | **Direct impact on maternal and child health** | **Data recency** | **Strength of data collection methodology** | **Representativeness of our target population** |  |
|  | | **1=Yes; 0=No** | **Scale 1 to 3. A score of 3 represents that the indicators is suggested by literature to be a proximal indicator of MCH health.** | **1=Data after 2016;**  **0=Data before 2016** | **1=Low quality;**  **2=High quality** | **1=Age or pregnancy is reflected in the indicator's denominator or nominator;**  **0=Not** |  |
| **Perinatal and Neonatal Outcomes Domain:** | |  |  |  |  |  |  |
|  | Late prenatal care | 0 | 3 | 1 | 2 | 1 | 7 |
|  | Preterm birth | 1 | 3 | 1 | 2 | 1 | 8 |
|  | Low birth weight | 1 | 3 | 1 | 2 | 1 | 8 |
|  | NICU admission | 0 | 2 | 1 | 2 | 1 | 6 |
|  | Late/no breastfeeding initiation | 0 | 3 | 1 | 1 | 1 | 6 |
|  | Infant mortality | 0 | 3 | 1 | 2 | 1 | 7 |
|  | Child mortality | 0 | 3 | 1 | 2 | 1 | 7 |
|  | Maternal depression | 0 | 3 | 1 | 2 | 1 | 7 |
|  | Well-baby visits | 0 | 3 | 1 | 2 | 1 | 7 |
|  | Young child well-child visit | 0 | 3 | 1 | 1 | 1 | 6 |
|  | Racial disparity in low birth weight | 0 | 3 | 1 | 2 | 1 | 7 |
| **Substance Use Domain:** | |  |  |  |  |  |  |
|  | Postpartum high-risk opioid use | 0 | 3 | 1 | 2 | 1 | 7 |
|  | Substance treatment facilities | 0 | 1 | 1 | 2 | 0 | 4 |
|  | Mental health treatment facilities | 0 | 2 | 1 | 2 | 0 | 5 |
|  | Buprenorphine physicians | 0 | 1 | 1 | 2 | 0 | 4 |
|  | Impaired drivers | 0 | 1 | 1 | 1 | 0 | 3 |
|  | Overdose deaths | 0 | 2 | 1 | 2 | 0 | 5 |
|  | Opioid overdose hospitalizations | 0 | 2 | 1 | 2 | 0 | 5 |
|  | Neonatal abstinence syndrome | 0 | 3 | 1 | 2 | 1 | 7 |
|  | Pregnancy and postpartum substance use disorder | 0 | 3 | 1 | 2 | 1 | 7 |
|  | Alcohol use disorder | 1 | 1 | 1 | 1 | 0 | 4 |
|  | Marijuana use | 1 | 1 | 1 | 1 | 0 | 4 |
|  | Cocaine use | 1 | 1 | 1 | 1 | 0 | 4 |
|  | Heroin use | 1 | 1 | 1 | 1 | 0 | 4 |
|  | Maternal smoking during pregnancy | 0 | 3 | 1 | 2 | 1 | 7 |
| **Socioeconomic Status Domain:** | |  |  |  |  |  |  |
|  | Poverty | 1 | 1 | 1 | 2 | 0 | 5 |
|  | Child poverty | 0 | 3 | 1 | 2 | 1 | 7 |
|  | Income inequality | 1 | 2 | 1 | 2 | 0 | 6 |
|  | Unemployment | 1 | 2 | 1 | 2 | 0 | 6 |
|  | Teens Not in School | 1 | 1 | 1 | 2 | 0 | 5 |
|  | Teen births | 0 | 3 | 0 | 2 | 1 | 6 |
|  | Mothers without high school diploma | 0 | 3 | 1 | 2 | 1 | 7 |
|  | Public assistance | 0 | 2 | 1 | 2 | 1 | 6 |
|  | Renters who are cost burdened | 0 | 2 | 1 | 2 | 0 | 5 |
|  | WIC redemptions | 0 | 3 | 0 | 2 | 1 | 6 |
|  | Child food insecurity | 0 | 3 | 1 | 2 | 1 | 7 |
| **Child Safety and Maltreatment Domain:** | |  |  |  |  |  |  |
|  | Child Maltreatment | 1 | 3 | 1 | 2 | 1 | 8 |
|  | Substantiated young child abuse and neglect | 0 | 3 | 1 | 2 | 1 | 7 |
|  | Abuse against pregnant and postpartum women | 0 | 2 | 1 | 1 | 1 | 5 |
|  | Domestic violence-related deaths among women of childbearing age | 0 | 2 | 1 | 2 | 0 | 5 |
|  | Protection from abuse order | 0 | 2 | 1 | 2 | 0 | 5 |
|  | Infant non-superficial injury | 0 | 3 | 0 | 2 | 1 | 6 |
|  | Young child non-superficial injury | 0 | 3 | 0 | 2 | 1 | 6 |
|  | Child welfare in-home services | 0 | 2 | 1 | 2 | 1 | 6 |
|  | Substance Use Need | 0 | 2 | 1 | 2 | 0 | 5 |
| **Community Environment Domain:** | |  |  |  |  |  |  |
|  | SNAP-authorized stores | 0 | 1 | 0 | 2 | 0 | 3 |
|  | WIC-authorized stores | 0 | 1 | 0 | 2 | 0 | 3 |
|  | Low-income and low-access census tracts | 0 | 2 | 0 | 2 | 0 | 4 |
|  | Hospitals | 0 | 1 | 1 | 2 | 0 | 4 |
|  | Community Health Centers | 0 | 3 | 1 | 2 | 0 | 6 |
|  | Primary care physicians | 0 | 3 | 1 | 2 | 0 | 6 |
|  | Pediatric Dentists | 0 | 1 | 1 | 2 | 0 | 5 |
|  | Crimes | 0 | 1 | 1 | 2 | 1 | 5 |
|  | Juvenile arrests | 1 | 1 | 1 | 2 | 0 | 5 |
|  | Environmental quality | 1 | 1 | 1 | 2 | 0 | 4 |
|  | Libraries | 0 | 2 | 0 | 2 | 0 | 4 |
|  | Public Transit in Urban Counties | 0 | 1 | 1 | 2 | 0 | 4 |
|  | Car Ownership in Rural Counties | 0 | 1 | 1 | 2 | 0 | 4 |
|  | Children Blood Lead Level | 0 | 1 | 1 | 2 | 0 | 7 |
|  | Residential Segregation | 0 | 3 | 1 | 2 | 1 | 5 |
| **Child Care Domain:** | |  |  |  |  |  |  |
|  | Regulated Child Care | 0 | 3 | 1 | 2 | 0 | 6 |
|  | High-quality Child Care | 0 | 3 | 1 | 2 | 0 | 6 |
|  | Subsidized Child Care | 0 | 3 | 1 | 2 | 1 | 7 |
|  | Publicly Funded Pre-K | 0 | 3 | 1 | 2 | 1 | 7 |
|  | Quality of Subsidized Child Care | 0 | 3 | 1 | 2 | 1 | 7 |
