# Supplementary material for: Integrating the Built and Social Environment into Health Assessments for Maternal and Child Health: Creating a Planning-Friendly Index
Source: Int J Environ Res Public Health. 2020 Dec 10;17(24):9224. doi: 10.3390/ijerph17249224 (PMC7763863; doi:10.3390/ijerph17249224)
Supplement: Supplementary file 1 [file ijerph-17-09224-s001.zip › supplementary 3.docx]

Supplementary 3

Includes detailed description on all indicators and data sources.

Description of Indicators and Data Sources

SOCIOECONOMIC STATUS DOMAIN

Poverty

Indicator Definition: Percent of population living below 100% Federal Poverty Level (FPL)

Data source: Census Small Area Income and Poverty Estimates

Link: <https://www.census.gov/programs-surveys/saipe/data/api.html>

The U.S. Census Bureau's Small Area Income and Poverty Estimates (SAIPE) program provides annual estimates of income and poverty statistics for all school districts, counties, and states.

Following the Office of Management and Budget's (OMB) Statistical Policy Directive 14, the Census Bureau uses a set of money income thresholds that vary by family size and composition to determine who is in poverty. If a family's total income is less than the family's threshold, then that family and every individual in it is considered in poverty. The official poverty thresholds do not vary geographically, but they are updated for inflation using Consumer Price Index (CPI-U). The official poverty definition uses money income before taxes and does not include capital gains or noncash benefits (such as public housing, Medicaid, and food stamps) (<https://www.census.gov/topics/income-poverty/poverty/about.html>)

Child poverty

Indicator definition: Percent of children under age 5 living below 100% Federal Poverty Level

Data source: Census Small Area Income and Poverty Estimates

Link: <https://www.census.gov/programs-surveys/saipe/data/api.html>

Income inequality

Indicator definition: Gini coefficient

Data source: American Community Survey

Link to data source methodology: <https://www.census.gov/newsroom/press-releases/2017/acs-single-year.html>

The ACS data provides demographic, social, economic and housing characteristic estimates on a rolling basis. The Gini index is a standard economic measure of income inequality. A score of 0.0 is perfect equality in income distribution. A score of 1.0 indicates total inequality where one household has all of the income.

Unemployment

Indicator definition: Unemployed percent of the civilian labor force

Data source: Bureau of Labor Statistics

Link: <https://www.bls.gov/lau/#cntyaa>

Teens not in school

Indicator definition: Percent of 16-19 year olds not enrolled in school with no high school diploma

Data source: American Community Survey

Link: https://factfinder.census.gov

Teen births

Indicator definition: Number of births per 1,000 female population ages 15-19

Data source: National Center for Health Statistics - Natality files

Link:<https://www.countyhealthrankings.org/app/pennsylvania/2019/measure/factors/14/data>

Public assistance

Indicator definition: Percent of households with children under 18 years who have received SSI, Cash Assist, or SNAP in the past 12 months

Data source: American Community Survey

Link: <https://www.census.gov/library/publications/2015/acs/acsbr13-08.html>

The ACS data provides demographic, social, economic and housing characteristic estimates on a rolling basis. The ACS question about SNAP identifies households in which one or more current members received SNAP during the past 12 months. Data reflect households, not individuals. If any person living at the sample address at the time of the interview received SNAP in the past 12 months, then the household is included in the estimate of SNAP participation.

Renters who are cost burdened

Indicator definition: Percent of households with cost burdens (paying more than 30 percent of their total household income for housing).

Data source: US Census, Federal Reserve Bank

Link: <https://www.policymap.com/widget?sid=107&wkey=362CA318B2A59032D42BAA70F6A23C4B>

WIC redemptions

Indicator definition: Per capita dollar amount of WIC redemptions

Data source: Department of Agriculture

Link: <http://www.policymap.com/our-data-directory.html#USDA%20Food%20Environment%20Atlas>

Child food insecurity

Indicator definition: Percent of children living in households that experienced food insecurity at some point during 2017

Data source: Feeding America

Link: <http://www.policymap.com/our-data-directory.html#Feeding%20America>

Food security measures the household-level economic and social condition of limited or uncertain access to adequate food. It is assessed in the Current Population Survey and represented in USDA food-security reports. The child food-insecurity estimates are derived from the same questions used by the USDA to identify food insecurity in households with children.

MATERNAL AND CHILD HEALTH DOMAIN

Late prenatal care

Indicator definition: Percent of births to mothers who did NOT initiate prenatal care in the first trimester

Data Source: Pennsylvania Vital Statistics annual report series

Link: https://datacenter.kidscount.org/data/tables/2732-prenatal-care--births-to-mothers-who-did-not-receive-early-prenatal-care#detailed

Preterm birth

Indicator definition: Percent of live births <37 completed gestational weeks

Data Source: National Vital Statistics System

Low birth weight

Indicator definition: Percent of live births <2500 grams at birth

Data Source: National Vital Statistics System

Neonatal Intensive Care Unit (NICU) admission

Indicator Definition: Percent of live births admitted to a neonatal intensive care unit

Data source: Definitions: Birth Records, Pennsylvania Department of Health, Bureau of Health Statistics and Research

NICU admission information was extracted from individual birth certificates. The county-level percent of NICU admissions among all live births was calculated by PolicyLab. For the purposes of birth certificate reporting, the CDC defines NICU admission as “admission into a facility or unit staffed and equipped to provide continuous mechanical ventilator support for the newborn.” (Birth Edit Specifications for the 2003 Proposed Revision of the U.S. Standard Certificate of Birth.http://www.cdc.gov/nchs/data/dvs/birth_edit_specifications.pdf.)

Late/no breastfeeding initiation

Indicator definitions: Percent of live births who were NOT breastfed at hospital discharge

Data source: Birth Records, Pennsylvania Department of Health, Bureau of Health Statistics and Research

Breastfeeding information was extracted from individual birth certificates. The county-level percent of late/no breastfeeding initiation among all live births was calculated by PolicyLab. In the birth certificate, breastfeeding status is assessed using the question: “Is the infant breastfed at discharge?” with the responses listed as “yes” or “no.”

Infant mortality

Indicator definition: Infant mortality rate per 1,000 live births

Numerator: county-level number of infant deaths in 2016 were accessed from PA Department of Health (<https://www.health.pa.gov/topics/HealthStatistics/VitalStatistics/DeathStatistics/Documents/Death_InfantAgeSexRaceYear_PA_2012_2016.pdf>)

Denominator: county-level number of live births in 2016 were accessed from PA Department of Health (<https://www.health.pa.gov/topics/HealthStatistics/VitalStatistics/BirthStatistics/Pages/birth-statistics.aspx>)

Child mortality

Indicator definition: Child death rate per 1,000 residents aged 0 to 4

Numerator: county-level numbers of child deaths under age 5 in 2016

Data source for numerator: Pennsylvania Department of Health

Link: <https://www.health.pa.gov/topics/HealthStatistics/VitalStatistics/DeathStatistics/Pages/death-statistics.aspx>

Denominator: county-level numbers of residents aged 0-4

Data source for denominator: U.S. Census of Population and Housing Unit 2016 Estimates

Link: [https://datacenter.kidscount.org/data/tables/9015-population--children-ages-0-19-by-race-and-age group#detailed/2/any/false/870,573,869,36,868,867,133/3008,3655,2318,4929,2320,2321,2322|577/18038,18039](https://datacenter.kidscount.org/data/tables/9015-population--children-ages-0-19-by-race-and-age%20group#detailed/2/any/false/870,573,869,36,868,867,133/3008,3655,2318,4929,2320,2321,2322|577/18038,18039)

Maternal depression

Indicator definition: Prevalence of diagnosed depression in the 2016 calendar year among Medicaid-enrolled women who were pregnant or gave birth during 2014-2016

Data source: Medicaid Claims, Pennsylvania Department of Human Services Office of Medical Assistance Programs, and Birth Certificate Records, Pennsylvania Department of Health, Bureau of Health Statistics and Research

Denominator: Pregnant women or those who gave birth during 2014-2016. This population was identified with a linkage of birth certificate records with Medicaid eligibility files.

Numerator: Women with presence of depression diagnosis in outpatient or inpatient encounter claims in the 2016 calendar year Depression was identified using International Classification of Diseases, 10th Revision (ICD-10) diagnostic codes.

Well-baby visits / Well-child visits

Indicator definition: Median number of well-child visits among Medicaid-enrolled children aged less than 1 year and children aged 1-5 years

Data source: Medicaid Claims, Pennsylvania Department of Human Services Office of Medical Assistance Programs

We identified Medicaid claims for well-child visits using International Classification of Diseases, 10th Revision (ICD-10) diagnostic codes and Current Procedural Terminology (CPT) codes in outpatient encounters.

The median number of well-child visits per child in each specified age range was calculated for each county.

Racial disparity in low birth weight

Indicator definition: Ratio of low-birth-weight rate in births born to Black mothers to low-birth-weight rate in births born to white mothers

Data Source: Birth Certificate Records, Pennsylvania Department of Health, Bureau of Health Statistics and Research

Link:<https://www.health.pa.gov/topics/HealthStatistics/VitalStatistics/BirthStatistics/Pages/birth-statistics.aspx>

Birth weight and mother’s race were obtained from birth certificates. Low-birth-weight rate is defined as percent of live births <2500 grams at birth.

SUBSTANCE USE DOMAIN

Postpartum high-risk opioid use

Indicator definition: Rate of mothers filling >=2 opioid prescriptions in the 2017 calendar year among Medicaid-enrolled mothers who delivered live births during 2015-2016

Data source: Medicaid Claims, Pennsylvania Department of Human Services Office of Medical Assistance Programs and Birth Certificate Records, Pennsylvania Department of Health, Bureau of Health Statistics and Research

Denominator: Women who delivered live birth during 2015-2016. This population were identified through linking birth certificate data with Medicaid eligibility file.

Numerator: We identified filled opioid prescriptions in pharmacy claims in 2017. Opioid medications were identified using GC3 therapeutic class codes (abbreviated Hierarchical Ingredient Code). Number of opioid prescriptions were calculated for each mother. High-risk opioid use is flagged as the presence of >=2 opioid prescriptions in the 2017 calendar year among new mothers.

Substance Treatment Facilities

Indicator definition: Number of substance treatment facilities per 100,000 residents

Data source: Substance Abuse and Mental Health Services Administration Behavioral Health Treatment Services Locator

Link: <https://findtreatment.samhsa.gov/locator>

The Behavioral Health Treatment Services Locator is a product of SAMHSA's Center for Behavioral Health Statistics and Quality (CBHSQ). The Locator is compiled from responses to the National Survey of Substance Abuse Treatment Services and the National Mental Health Services Survey.

The Behavioral Health Treatment Services Locator displays the street address at which services are provided. Organizations delivering services at different locations are requested to supply information about the services at each location, although a central intake number may be displayed if requested.

Eligible substance abuse and addiction treatment facilities include:

1. Licensure/accreditation/approval to provide substance abuse treatment from the state substance abuse agency (SSA) or a national treatment accreditation organization (e.g., The Joint Commission, CARF, NCQA, etc.)
2. Staff who hold specialized credentials to provide substance abuse treatment services
3. Authorization to bill third-party payers for substance abuse treatment services using an alcohol or drug client diagnosis

Mental health treatment facilities

Indicator definition: Number of mental health treatment facilities per 100,000 residents

Data source: Substance Abuse and Mental Health Services Administration (SAMHSA) Behavioral Health Treatment Services Locator

Link: <https://findtreatment.samhsa.gov/locator>

Eligible mental health treatment facilities include:

1. Facilities that provide mental health treatment services and are funded by the state mental health agency (SMHA) or other state agency or department
2. Mental health treatment facilities administered by the U.S. Department of Veterans Affairs
3. Private for-profit and non-profit facilities that are licensed by a state agency to provide mental health treatment services, or that are accredited by a national treatment accreditation organization (e.g., The Joint Commission, NCQA, etc.)
4. Facilities that are not eligible for the Behavioral Health Treatment Services Locator include:
5. Facilities that provide either mental health or substance abuse treatment exclusively to persons who are incarcerated
6. Facilities whose primary or only focus is the provision of services to persons with Mental Retardation (MR), Developmental Disability (DD), or Traumatic Brain Injuries (TBI)
7. Mental health professionals in private practice (individual) or in a small group practice not licensed or certified as a mental health clinic or (community) mental health center

Buprenorphine physicians

Indication definition: Number of Buprenorphine treatment practitioner per 100,000 residents

Data source: Substance Abuse and Mental Health Services Administration (SAMHSA)

Link: <https://www.samhsa.gov/medication-assisted-treatment/training-materials-resources/physician-program-data>

SAMHSA evaluates the buprenorphine waiver program under the Drug Addiction Treatment Act of 2000 (DATA 2000) and tracks the number of DATA-certified practitioners. SAMHSA uses the Online Request for Patient Limit Increase to implement the provisions of DATA 2000 that permit physicians to prescribe or dispense buprenorphine.

Impaired drivers

Indicator definition: Number of vehicle crashes involving impaired driver per 100,000 residents

Data source: Pennsylvania Department of Transportation (PennDOT)

Link: <https://pennshare.maps.arcgis.com/apps/webappviewer/index.html?id=8fdbf046e36e41649bbfd9d7dd7c7e7e>

Crash data is derived from the information that comes from a reportable crash. A reportable crash according to Title 75, Pennsylvania Consolidated Statutes, Section 3746(a) is: An incident that occurs on a highway or traffic way that is open to the public by right or custom and involved at least one motor vehicle in transport. An incident is reportable if it involves: 1) Injury to or death of any person, or 2) Damage to any vehicle to the extent that it cannot be driven under its own power in its customary manner without further damage or hazard to the vehicle, other traffic elements, or the roadway, and therefore requires towing; 3) Crash data does not include non-reportable crashes or near misses; 4) Crash data may not contain complete information, some elements may be unknown.

Data obtained from the PennDOT Open Data Portal are CSV files. These files are:

1. “CRASH”: Information about the crash such as: 1) Where: County, Municipality, Work zone; 2) When: Date, Time, Day of Week, Hour of Day, Month of Year; 3) Item Counts: People, Vehicles, Unbelted, Fatal, etc.
2. “FLAG”: Series of Yes/No items that help refine lookups for specific factors about the crash such as: Drinking Driver, Use of a Cell Phone, Fatal Crash, Motorcycle involved, and over 60 other crash defining items.

Overdose deaths

Indicator definition: Rate of overdose deaths per 100,000 people aged 15-64 years

Data source: OverdoseFreePA

Link: <https://www.overdosefreepa.pitt.edu/wp-content/uploads/2018/10/PA-Opioid-Report-Final.pdf>

As in 2015 and 2016, the Drug Enforcement Administration (DEA) Philadelphia Field Division (PFD) requested information on drug-related overdose deaths from Pennsylvania’s coroners and medical examiners for deaths that occurred in 2017. In 2017, Pennsylvania coroners and medical examiners reported 5,456 drug-related overdose deaths (ruled accidental or undetermined). This number represents a rate of 43 deaths per 100,000 people, ranged from 0 to 77 among individual counties. Between 2015 and 2017, there was a 65% increase in the number of drug-related overdose deaths in Pennsylvania.

OverdoseFreePA.org is operated by the Pennsylvania Overdose Prevention Technical Assistance Center (TAC) and is a collaboration between the TAC, Pennsylvania communities, and six partner organizations. The TAC is based out of the Program Evaluation and Research Unit (PERU) at the University of Pittsburgh’s School of Pharmacy.

Opioid overdose hospitalizations

Indicator definition: Rate of hospitalizations for opioid overdose (heroin and opioid pain medicine combined) per 100,000 residents age 15 and above

Data source: Pennsylvania Health Care Cost Containment Council (PHC4)

Link: <https://data.pa.gov/Opioid-Related/Rate-of-Hospitalizations-for-Opioid-Overdose-per-1/3f26-q827>

This analysis is restricted to Pennsylvania residents age 15 and older who were hospitalized in Pennsylvania general acute care hospitals.

Neonatal abstinence syndrome

Indicator definition: Rate of Neonatal Abstinence Syndrome (NAS) per 1000 newborn hospital stays

Data source: Pennsylvania Health Care Cost Containment Council (PHC4)

Link: <https://data.pa.gov/Opioid-Related/Rate-of-Neonatal-Abstinence-Syndrome-per-1-000-New/2ats-ttun>

Countywide counts of newborn hospital stays with Neonatal Abstinence Syndrome (NAS) and countywide rates of newborn hospital stays with (NAS) per 1,000 newborn stays. Neonatal Abstinence Syndrome, or neonatal drug withdrawal, is an array of problems that develops shortly after birth in newborns who were exposed to addictive drugs, most often opioids, while in the mother’s womb. Withdrawal signs develop because these newborns are no longer exposed to the drug for which they have become physically dependent. This analysis is restricted to newborns with Pennsylvania-state residence who were hospitalized in Pennsylvania hospitals.

Pregnancy and postpartum substance use disorder

Indicator definition: Rate of diagnosed substance use disorder in the 2016 calendar year among Medicaid-enrolled mothers who were pregnant or delivered live births during 2014-2016

Data source: Medicaid Claims, Pennsylvania Department of Human Services Office of Medical Assistance Programs and Birth Certificate Records, Pennsylvania Department of Health, Bureau of Health Statistics and Research

Denominator: Pregnant women or women who gave live birth in 2014-2016 . This population was identified using a linkage of birth certificate records with Medicaid eligibility files.

Numerator: Diagnosis of substance use disorder was identified using International Classification of Diseases, 10th Revision (ICD-10) diagnostic codes from inpatient and outpatient encounters.

Alcohol use disorder

Indicator definition: Prevalence rate of Alcohol Use Disorder among individuals aged 12 and older

Data source: Substance Abuse and Mental Health Services Administration (SAMHSA) - National Survey of Drug Use and Health

Link: <https://www.samhsa.gov/data/report/2017-methodological-summary-and-definitions>

The National Survey on Drug Use and Health (NSDUH) provides estimates of substance use and mental illness at the national, state, and sub-state levels.

NSDUH collects data through face-to-face interviews with residents of households who are: US civilians; older than 12 years old; not institutionalized. The Substate Reports use three years of combined NSDUH data (with one year overlapping) and provide state estimates for a subset of measures of substance use and mental health outcomes. These estimates are based on an small area estimation (SAE) methodology in which substate-level NSDUH data are combined with county and census block group and tract-level data from the 50 states and the District of Columbia. Definitions for Pennsylvania sub-state region in NSDUH 2014-2016 can be found at <https://www.samhsa.gov/data/report/2014-2016-nsduh-substate-region-definitions>

Marijuana use

Indicator definition: Prevalence rate of marijuana use in the month before survey completion among individuals aged 12 and older

Data source: Substance Abuse and Mental Health Services Administration (SAMHSA) - 2014-2016 National Survey of Drug Use and Health

Measures of use of marijuana in the respondent's lifetime, the past year, and the past month were derived from responses to the question about recency of use: "How long has it been since you last used marijuana or hashish?" The question about recency of use was asked if respondents previously reported any use of marijuana or hashish in their lifetime. Responses to separate questions about use of cigars with marijuana in them (blunts) were not included in these measures. Creation of these measures did not take into account responses to questions that have been included in the survey since 2013 about use of marijuana in the past 12 months that was recommended by a doctor or other health care professional.

Cocaine use

Indicator definition: Prevalence rate of cocaine use in the year before survey completion among individuals aged 12 and older

Data source: Substance Abuse and Mental Health Services Administration (SAMHSA) – 2014-2016 National Survey of Drug Use and Health

Measures of use of cocaine, including powder, crack, free base, and coca paste, in the respondent's lifetime, the past year, and the past month were derived from responses to the question about recency of use: "How long has it been since you last used any form of cocaine?" The question about recency of use was asked if respondents previously reported any use of cocaine in their lifetime.

Heroin use

Indicator definition: Prevalence rate of heroin use in the year before survey completion among individuals aged 12 and older

Data source: Substance Abuse and Mental Health Services Administration (SAMHSA) 2014-2016 National Survey of Drug Use and Health

Measures of use of heroin in the respondent's lifetime, the past year, and the past month were derived from responses to the question about recency of use: "How long has it been since you last used heroin?" The question about recency of use was asked if respondents previously reported any use of heroin in their lifetime.

Maternal smoking during pregnancy

Indicator definitions: Percent of children born to mothers who used tobacco during pregnancy

Data Source: Pennsylvania Vital Statistics annual report series

Link: [https://datacenter.kidscount.org/data/tables/2734-tobacco-use--births-to-mothers-who-used-tobacco-during-pregnancy](https://datacenter.kidscount.org/data/tables/2734-tobacco-use--births-to-mothers-who-used-tobacco-during-pregnancy#detailed/5/5379-5445/false/573,869,36,868,867,133,38,35,18,17/any/5672,9347)

CHILD SAFETY AND MALTREATMENT DOMAIN

Child maltreatment

Indicator definition: Number of substantiated child maltreatment victims under 18 years old

Data source: Pennsylvania Department of Human Services, Office of Children, Youth and Families (OCYF)

Link: <https://www.dhs.pa.gov/docs/Publications/Documents/2019%20child%20prev.pdf>

Child protective services reports are those that allege a child might have been a victim of child abuse and are made by mandated and permissive reporters. In PA, the county children and youth agencies (CCYA) immediately begins an investigation upon receipt of a report of suspected. A child abuse investigation must determine within 30 days whether the report is: 1) “founded” (there is court action, including: a judicial adjudication that the child was abused, acceptance into an accelerated rehabilitative disposition program, consent decree entered in a juvenile proceeding; or granting of a final protection from abuse order); 2) “Indicated” (CCYA or OCYF Regional Office staff find substantial evidence that abuse has occurred based on medical evidence, the child protective service investigation, or an admission by the perpetrator); 3) “Unfounded” (the alleged abuse did not meet the definition or criteria for abuse); or 4) “Pending”. In OCYF data, “founded” and “indicated” reports of child abuse are referred to as “substantiated” reports.

Substantiated young child abuse and neglect

Indicator definition: Number of substantiated child abuse and neglect per 1000 children aged 0-4

Data source: Pennsylvania Department of Human Services, Office of Children, Youth and Families

Substantiated abuse includes: children for whom a judge has found abuse and children determined to have experienced abuse based on medical evidence, the child protective services investigation, or an admission by the perpetrator.

Abuse against pregnant and postpartum women

Indicator definition: Rate of diagnosed abuse in the 2016 calendar year among Medicaid-enrolled pregnant women or women who gave live birth during 2014 - 2016

Data source: Medicaid Claims, Pennsylvania Department of Human Services Office of Medical Assistance Programs and Birth Certificate Records, Pennsylvania Department of Health, Bureau of Health Statistics and Research

Denominator: Pregnant women or women who gave birth in 2014-2016 . This population was identified using a linkage of birth certificate records with Medicaid eligibility files.

Numerator: Diagnosis of adult abuse was identified using International Classification of Diseases, 10th Revision (ICD-10) diagnostic codes in inpatient and outpatient encounters. Diagnoses included: 1) adult physical /sexual /psychological abuse or neglect, 2) encounter for mental health services for victim of spousal or partner abuse, and 3) physical abuse complicating pregnancy, childbirth and the puerperium.

Domestic violence-related deaths among women of childbearing age

Indicator definition: Number of domestic violence-related deaths per 1000 female aged 15-50 years

Data source: Pennsylvania Coalition against Domestic Violence (PCADV)

Link: <https://www.pcadv.org/wp-content/uploads/2018-Fatality-Report_web.pdf>

PCADV compiles its annual list based on news accounts, police departments, and information received from 59 local domestic violence programs serving all 67 counties. PCADV uses a conservative method of identifying domestic violence-related deaths. The death count does not include cases where no arrests have been made or where the relationship between the victim and perpetrator is unclear.

The numerator of this indicator is number of victims that have the following characteristics: 1) female; 2) relationship to perpetrator: girlfriend; ex-girlfriend; wife; current intimate partner; former intimate partner; domestic violence-related; 3) aged 15-50 years at the time of death. The denominator of this indicator is number of female residents aged 15-50 years in each county, based on data from census.

Protection from abuse order

Indicator definition: Number of judge-grated protection from abuse order per 1000 residents

Data source: PA Courts

Link: <http://www.pacourts.us/news-and-statistics/research-and-statistics/dashboard-table-of-contents/protection-from-abuse>

The numerator of this indicator is the number of final orders on protection from abuse petition following an evidentiary hearing before a judge by county. The denominator is the total population in each county.

Infant and young child non-superficial injury

Indicator definition: Prevalence of children having non-superficial injury during the first year of life (for infant) or during the first 5 years of life (for young child) per 1000 Medicaid-enrolled children

Data source: Medicaid Claims, Pennsylvania Department of Human Services Office of Medical Assistance Programs

Diagnosis of non-superficial injury was identified using International Classification of Diseases, 9th Revision (ICD-9) codes in children’s outpatient and inpatient encounters. Non-superficial injuries include: dislocation, fracture, and crush injuries, poisonings, and burns. Car accident injuries were excluded.

Substance use need

Indicator definition: Composite need score of a set of substance use disorder related indicators

Data source: Multiple sources (please see Substance Use Domain for details)

PolicyLab derived a composite score that summarizes the overall need level of a set of indicators in the substance use domain, including: Postpartum high-risk opioid use; Impaired drivers; Overdose deaths; Opioid overdose hospitalization; Neonatal abstinence syndrome; Pregnancy and Postpartum Substance Use Disorder; Alcohol use disorder; Marijuana use; Cocaine use; Heroine use; and Maternal smoking during pregnancy.

Child welfare in-home services

Indicator definition: Percent of children under 18 receiving child welfare in-home services

Data source: Pennsylvania Department of Human Services, Office of Children, Youth and Families

The numerator of this indicators is number of children who received services provided to both parents and children to address concerns related to child safety and well-being to enable the children to remain safely in their own home by county. This includes children whose families were screened-in and/or accepted for services. The denominator is number of children under 18 years old in each county.

COMMUNITY AND ENVIRONMENT DOMAIN

SNAP-authorized stores

Indicator definition: number of SNAP authorized stores per 1000 families

Numerator: Number of SNAP authorized stores in each county

Data source for numerator: USDA

Link: <https://www.ers.usda.gov/data-products/food-environment-atlas/go-to-the-atlas/>

Supplemental Nutrition Assistance Program (SNAP) benefits are issued to qualifying low-income individuals to supplement their ability to purchase food. Eligibility is determined by state authorities’ interpretations of Federal regulations. The U.S. Department of Agriculture (USDA) pays the cost of the assistance. Data on the Supplemental Nutrition Assistance Program (SNAP), formerly known as the Food Stamp Program, come from the Food and Nutrition Service of USDA’s SNAP Benefits Redemption Division. The SNAP Retailer Locator at the USDA website contains a list of all retailers that accept SNAP payments (sometimes known as food stamps).

Denominator: Number of families with children

Data source for denominator: Census

WIC-authorized stores

Indicator definition: number of WIC authorized stores per 1000 families with children under age 6

Numerator: Number of WIC authorized stores in each county

Data source for numerator: United States Department of Agriculture (USDA)

Link: <https://www.ers.usda.gov/data-products/food-environment-atlas/go-to-the-atlas/>

Data on the Women, Infants, and Children (WIC) program come from the Food and Nutrition Service of USDA’s Program Analysis and Monitoring Branch, Supplemental Food Programs Division.

Denominator: Number of children under 6

Data source for denominator: Census

Low income and low access census tracts

Indicator definition: Percent of census tract with low income and low access

Data source: United States Department of Agriculture (USDA)

Link: <https://www.ers.usda.gov/data-products/food-access-research-atlas/documentation/>

The Food Access Research Atlas is a project of the Economic Research Service, the economic information and research division of the U.S. Department of Agriculture. The Atlas contains data about food access and can be used for determining eligibility for HFFI funds.

Low access to healthy food is defined as being far from a supermarket, supercenter, or large grocery store. A census tract has low access status if a certain number of share of individuals in the tract live far from a supermarket. There are various measures for distance from a supermarket that this data uses. The original Food Desert Locator (which this replaces) defined low access as living 1 mile away from a supermarket in urban areas, and 10 miles away in rural areas. This study adds measures for 0.5 miles in urban areas, and 20 miles in rural areas. Using these distance measurements, a census tract is defined as low access if there are at least 500 people or 33 percent of the population within the tract with low access.

Low-income tracts are defined as where the tract’s poverty rate is greater than 20 percent, the tract’s median family income (MFI) is less than or equal to 80 percent of the statewide MFI, or the tract is in a metropolitan area and has an MFI less than or equal to 80 percent of the metropolitan area’s MFI.

Hospitals

Indicator definition: Number of hospital beds per 1,000 residents

Data source: HRSA

Link: <https://data.hrsa.gov/tools/data-explorer>

Data on Hospital are from the HRSA Geospatial Database. These geocoded locations from the HRSA Geospatial Data Warehouse are from a “Provider of Service” extract from the Online Survey and Certification Reporting System database maintained by Centers for Medicare and Medicaid Services. They are included in the HRSA Warehouse because they are the most readily-obtainable data on various classes of health care facility such as hospitals, hospices, rural health clinics, etc. The Hospitals are those facilities participating in Medicare and Medicaid Services for individuals requiring temporary or long-term medical treatment.

Community health centers

Indicator definition: Number of community health centers, FQHCs, and look alikes per 100,000 residents

Data source: HRSA

Link: <https://data.hrsa.gov/tools/data-explorer>

This dataset includes Federally Qualified Health Centers (FQHCs) that are receive funding under the Health Center Cluster federal grant program to provide care for underserved populations. The types of providers eligible include Community Health Centers, Migrant Health Centers, Health Care for the Homeless Programs, Public Housing Primary Care Programs, and care providers for some tribal organizations.

This data set also includes the community health centers that are eligible but not currently receiving grant funding. Although they are not receiving grants, these providers – or “look-alikes” – are eligible for some benefits including enhanced reimbursement from Medicare and Medicaid. Mapping both FQHCs and “look-alikes” might provide a fuller picture of the health-care safety net in a community.

Primary care physicians

Indicator definition: Number of primary care physicians per 1,000 residents

Data source: HRSA

Link: <https://data.hrsa.gov/topics/health-workforce/ahrf>

Primary care physicians included in this data are physicians (both MDs and DOs) who are providing hospital patient care. MDs and DOs are included all physicians and residents who are providing patient care in the fields of Family Medicine, Internal Medicine, General Practice, Surgery, Ob-Gyn, and Pediatrics.

Pediatric dentists

Indicator definition: Number of active clinical pediatric dentists per 1,000 children under age 18

Data source: American Dental Association (shared by PA Coalition for Oral Health)

Crimes

Indicator definition: Number of reported crimes per 1,000 residents

Data source: Institute for Social Research - National Archive of Criminal Justice Data

Juvenile arrests

Indicator definition: Number of crime arrests ages 0-17 per 100,000 juveniles aged 0-17

Data source: Institute for Social Research - National Archive of Criminal Justice Data

Libraries

Indicator definition: Number of libraries per 100,000 residents

Data source: Institute of Museum and Library Services (IMLS)

The Public Libraries Survey (PLS) is conducted annually by the IMLS. The data file includes all public libraries and outlets identified by state library agencies. Library outlet locations include central libraries, branches, and bookmobiles. Points were geocoded by IMLS based on addresses provided by the survey respondent (library administrator).

The original data provides geocoded locations of libraries. To aggregate the data to the county level, PolicyLab calculated the number of libraries in each county. To account for the difference in population size, we added the total number of residents as the denominator and used the “number of libraries per 100,000 residents” as the indicator.

Public transit in urban counties

Indicator definition: performance score in 6 urban counties (Delaware, Chester, Montgomery, Bucks, Philadelphia, and Allegheny)

Data source: AllTransit

Link: <https://alltransit.cnt.org/methods/>

AllTransit is a broad transit database developed and assembled by the Center for Neighborhood Technology (CNT) and designed to comply with the General Transit Feed Specification (GTFS).

AllTransit Metrics:

- Transit Connectivity Index (TCI): The TCI is an index scaled by the number of transit trips the average household in a block group can access by walking each week. The TCI is a measure of how connected the average household member is to the availability of a transit ride. This is a placed-based measure that is derived by examining the proximity of all transit routes, and the area covered by each route at different distances.
- Performance Score: it has values from 0 to 10, where the higher the number the better the transit service. It is a weighted sum of transit connectivity (TCI), access to land area and jobs, and frequency of service. It combines with the TCI, described above, that measures “can I get transit” with the Jobs Accessible in 30 Minute Transit Ride, which measures “what can I get to once I am on transit,” and combines them in a way that is reflective of what fraction of people use transit for a given type of trip – their journey to work – answering the question “how should I get to work.”

Car ownership in rural counties

Indicator definition: Percent of census tracts with low car ownership in 61 rural counties

Data source: American Community Survey

Link: <https://factfinder.census.gov/faces/nav/jsf/pages/download_center.xhtml>

ACS asks one question about how many vehicles are available in a household to understand access to transportation. The answers to this question were compiled by CDC as percentage of households in each census tract with no vehicle available. Policylab then described the distribution of this percentage in all census tracts in Pennsylvania rural areas. We defined census tract with low car ownership as a census tract with more than 6% (median in census tracts in PA rural area) of households with no vehicle available. We then further calculated the percent of census tracts with low car ownership in each county.

Environmental quality

Indicator definition: Average index score of potential exposure to harmful toxins

Data source: National Air Toxics Assessment (NATA) data

Link: <https://www.arcgis.com/home/item.html?id=8d292db7263c44eea5064186a91229ff>; <https://earthworks.stanford.edu/catalog/stanford-qh915ct6967>

The environmental health hazard exposure index summarizes potential exposure to harmful toxins at a neighborhood level. Potential health hazards exposure is a linear combination of standardized EPA estimates of air quality carcinogenic (c), respiratory (r) and neurological (n) hazards. Values are inverted and then percentile ranked nationally. Values range from 0 to 100. The higher the index value, the less exposure to toxins harmful to human health. Therefore, the higher the value, the better the environmental quality of the area.

To aggregate the data to county level, PolicyLab used the median of index scores of census tracts within each county to represent the average environmental hazard exposure of each county.

Children blood lead level

Indicator definition: Percent of tested children with confirmed elevated blood lead level

Data source: CDC National Childhood Blood Lead Surveillance Data

Link: <https://www.cdc.gov/nceh/lead/data/national.htm>

CDC’s childhood blood lead surveillance system at the national-level integrates information collected by state and local health departments. CDC applies nationally consistent standard definitions and classifications for blood lead surveillance data from all states: confirmed elevated blood lead level is defined as a child with one venous blood test ≥ 5 μg/dL or two capillary blood tests ≥ 5 μg/dL drawn within 12 weeks of each other. In addition, CDC applies rigorous error-checking and validation algorithms to the data submitted to ensure only one test per individual per year is counted. (Therefore, the information available from CDC will not match data reports from the individual states because states may use different case definitions for clinical and environmental management.)

In this project, we used percent of children with elevated BLLs as the indicator: the number of children less than 72 months of age with a confirmed elevated blood lead level divided by the number of children less than 72 months of age tested for blood

Residential segregation

Indicator definition: Index of dissimilarity where higher values indicate greater residential segregation between Black and White county residents.

Data source: Census; County Health Ranking

Link: <https://www.countyhealthrankings.org/app/pennsylvania/2020/measure/factors/141/data>

Residential segregation of Black and White residents is considered a fundamental cause of health disparities in the US and has been linked to poor health outcomes, including mortality, a wide variety of reproductive, infectious, and chronic diseases, and other adverse conditions. Structural racism is also linked to poor-quality housing and disproportionate exposure to environmental toxins. Individuals living in segregated neighborhoods often experience increased violence, reduced educational and employment opportunities, limited access to quality health care and restrictions to upward mobility. Racial/ethnic residential segregation refers to the degree to which two or more groups live separately from one another in a geographic area. The index of dissimilarity is a demographic measure of the evenness with which two groups (Black and White residents, in this case) are distributed across the component geographic areas (census tracts, in this case) that make up a larger area (counties, in this case).

The residential segregation index ranges from 0 (complete integration) to 100 (complete segregation). The index score can be interpreted as the percentage of either Black or White residents that would have to move to different geographic areas in order to produce a distribution that matches that of the larger area. A missing value is reported for counties with Black population less than 100 in the time frame.

CHILD CARE DOMAIN

Regulated childcare

Indicator definition: Number of regulated child care providers per 100 children residents under 3 years old

Data source: Pennsylvania Department of Education’s (PDE) Office of Child Development and Early Learning (OCDEL)

The regulated providers include center, group, and family child care. The number of providers does not include private licensed nursery schools, head start providers, or school districts offering pre-k.

High-quality childcare

Indicator definition: Percent of regulated child care providers meeting high-quality standards

Data source: Pennsylvania Departments of Education and Human Services, Office of Child Development and Early Learning. The percent is calculated by dividing the number of child care providers with Keystone STAR 3 or 4 designation by the number of total regulated providers.

Subsidized childcare

Indicator definition: Percent of children 0-5 eligible for Child Care Works (CCW) who were served by CCW

Data source: Pennsylvania Departments of Education and Human Services, Office of Child Development and Early Learning.

Child care subsidies are available to low-income working families to increase the opportunity for parents to obtain high-quality child care for their children while they work. The percent is calculated by the number and infants, toddlers and preschool-age children enrolled through the Child Care Works subsidized child care system divided by the number of children under 5 years who are eligible (i.e. have all available parents in the labor force and live below 200% poverty).

Quality of subsidized childcare

Indicator definition: Percent of children aged 0-5 receiving subsidized child care in Keystone STARS 3 or 4 facilities

Data source: Pennsylvania Departments of Education and Human Services, Office of Child Development and Early Learning.

The percent is calculated by the number and infants, toddlers and preschool-age children receiving subsidized child care in Keystone STARS 3 or 4 facilities divided by the total number of children under 5 years enrolled through the Child Care Works subsidized child care system

Publicly-funded Pre-K

Indicator definition: Percent of children aged 3-4 below 300% poverty with access to publicly funded, high-quality pre-k

Data source: Pennsylvania Departments of Education and Human Services, Office of Child Development and Early Learning.

Publicly funded, high-quality pre-k includes the distinct count of PA Pre-K Counts, Head Start Supplemental Assistance Program and Child Care Works enrollments in Keystone STARS 3 and 4; Head Start; and school district pre-k.
