# Supplementary material for: Integrating the Built and Social Environment into Health Assessments for Maternal and Child Health: Creating a Planning-Friendly Index
Source: Int J Environ Res Public Health. 2020 Dec 10;17(24):9224. doi: 10.3390/ijerph17249224 (PMC7763863; doi:10.3390/ijerph17249224)

Supplementary file 4 Correlations between need indices of six domains

For each of the six domains, a county’s domain composite need score is calculated as the weighted average of the need scores of the indicators within that domain. Domain need score was then categorized into need index using a quartile-based method:

- Domain need index = 1: if a county was categorized as having “low need” in a domain if the county’s composite need score ranked within the bottom 25% of all Pennsylvania counties
- Domain need index = 2: if a county was categorized as having “medium need” in a domain if the county’s composite need score ranked between the 25th and 75% percentiles of all Pennsylvania counties
- Domain need index = 3: if a county was categorized as having “elevated need” in a domain if the county’s composite need score ranked within the top 25% of all Pennsylvania counties

The below figure shows the spearman correlations between the need indices of the 6 domain. Overall, patterns of correlated risks were not strongly identifiable. Each county’s profile of risk and strength has unique components.


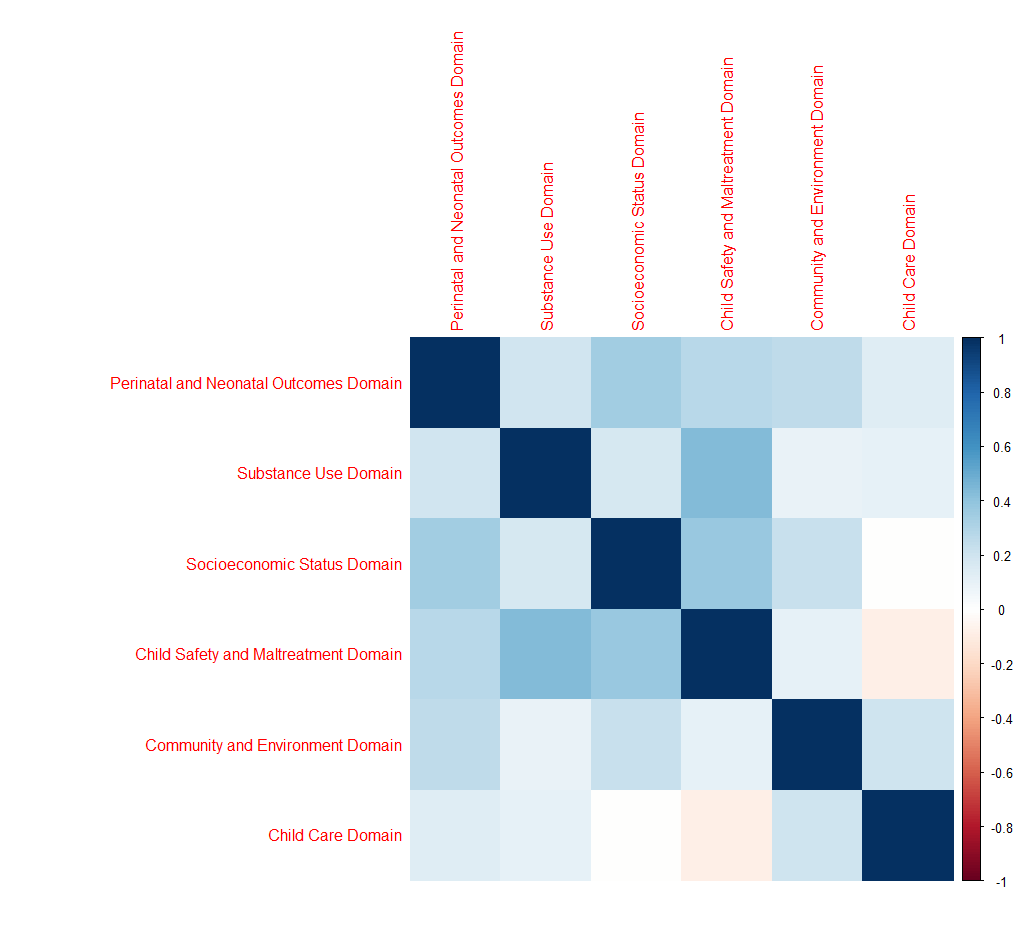

Supplement: Supplementary file 1 [file ijerph-17-09224-s001.zip › supplementary 4.docx]
